# Assessment of the Global Variance Effective Size of Subdivided Populations, and Its Relation to Other Effective Sizes

**DOI:** 10.1007/s10441-023-09470-w

**Published:** 2023-07-17

**Authors:** Ola Hössjer, Linda Laikre, Nils Ryman

**Affiliations:** 1 Division of Mathematical Statistics, Department of Mathematics, Stockholm University, 106 91 Stockholm, Sweden; 2 Division of Population Genetics, Department of Zoology, Stockholm University, 106 91 Stockholm, Sweden

**Keywords:** Genetic diversity, Length of time interval, Matrix analytic recursions, Metapopulation, Migration–drift equilibrium, Perturbation theory of matrices, Variance effective size, 60J28, 92D10, 92D15, 92D20

## Abstract

The variance effective population size ($N_{eV}$) is frequently used to quantify the expected rate at which a population’s allele frequencies change over time. The purpose of this paper is to find expressions for the global $N_{eV}$ of a spatially structured population that are of interest for conservation of species. Since $N_{eV}$ depends on allele frequency change, we start by dividing the cause of allele frequency change into genetic drift within subpopulations (*I*) and a second component mainly due to migration between subpopulations (*II*). We investigate in detail how these two components depend on the way in which subpopulations are weighted as well as their dependence on parameters of the model such a migration rates, and local effective and census sizes. It is shown that under certain conditions the impact of *II* is eliminated, and $N_{eV}$ of the metapopulation is maximized, when subpopulations are weighted proportionally to their long term reproductive contributions. This maximal $N_{eV}$ is the sought for global effective size, since it approximates the gene diversity effective size $N_{eGD}$, a quantifier of the rate of loss of genetic diversity that is relevant for conservation of species and populations. We also propose two novel versions of $N_{eV}$, one of which (the backward version of $N_{eV}$) is most stable, exists for most populations, and is closer to $N_{eGD}$ than the classical notion of $N_{eV}$. Expressions for the optimal length of the time interval for measuring genetic change are developed, that make it possible to estimate any version of $N_{eV}$ with maximal accuracy.

## Introduction

### Background on Effective Population Sizes

The effective population size $N_e$ is a well known concept (Wright 1931, 1938) that quantifies the rate at which genetic variation of a population is lost over time. This is important in conservation biology, where retention of sufficient levels of genetic diversity to allow adaptation to changing environmental conditions is of major concern for the long term viability and conservation of species and populations (Frankham et al. 2010; Traill et al. 2010; Hoban et al. 2021; Allendorf et al. 2022). Since many populations exhibit some type of geographic substructure, it is crucial to assess in which way and how much this impacts $N_e$. Typically, such a structure is modelled as a metapopulation that consists of a number of more or less connected subpopulations. For short term conservation of species it is mainly genetic drift within and migration between subpopulations that impact $N_e$, whereas mutation and natural selection are usually ignored.

Many versions of effective size have been proposed, as recently discussed by Gilbert and Whitlock (2015), Wang (2016), Waples (2016), Ryman et al. (2019), and Nadachowska-Brzyska et al. (2022). In this paper we focus on the variance effective size $N_{eV}$ (Crow 1954), where loss of genetic variation is quantified in terms of the variance of frequency change of genetic variants (alleles). If genetic data is available from at least two points in time, the temporal method (Kimbras and Tsakas 1971; Nei and Tajima 1981; Pollack 1983; Waples 1989; Jorde and Ryman 2007) can be employed to estimate $N_{eV}$. For this reason $N_{eV}$ is one of the most frequently used notions of effective size that is recommended because it is multigenerational (Frankham et al. 2019; Frankham 2021). On the other hand, $N_{eV}$ is typically *not* the best effective size for assessing the rate at which genetic diversity is lost in substructured populations. Since this rate is an important criterion for conservation of species, this is potentially a drawback of $N_{eV}$ (Ryman et al. 2019).

In order to find out whether versions of $N_{eV}$ for substructured populations exist that are more appropriate for estimating $N_e$ for conservation purposes, a first step is to understand how various parameters of a population genetic model influence $N_{eV}$. To this end, it is important to build a mathematical framework for how the genetic makeup of a population evolves over time, and then find expressions for the variance of allele frequency change. Using such an approach, Whitlock and Barton (1997) noted that $N_{eV}$ is a function of several parameters, such as the local effective sizes of subpopulations under isolation, the migration pattern between subpopulations and the way in which subpopulations are weighted in order for $N_{eV}$ to reflect, for instance, local or global aspects of the variance effective size. Hössjer et al. (2014) added local census sizes to the model and considered subpopulation weights of general form. Hössjer et al. (2016) noticed that previous analyses of $N_{eV}$ had been overly simplistic and neglected the impact of subpopulation differentiation at the first time point at which genetic data is collected.

### Objectives

The purpose of this paper is to find versions of $N_{eV}$ for the metapopulation that are of interest for conservation, by appropriately quantifying the rate of loss of genetic diversity. To this end, we will generalize work of Whitlock and Barton (1997), Ryman et al. (2014) and Hössjer et al. (2014, 2016) and study the variance effective size of structured populations by means of matrix analytic methods, where standardized covariances of allele frequency change and gene diversities (Nei 1973) are updated recursively over time. More specifically we consider three aspects of $N_{eV}$: (i) A careful analysis of allele frequency change and subpopulation weights, which will lead us to a version of $N_{eV}$ that is of interest for conservation, (ii) Introduction of two novel and more stable ways of defining $N_{eV}$, both of which have versions that are of relevance for conservation, (iii) Finding expressions for the length of the interval between the two time points at which genetic data is collected, which is optimal in terms of estimating $N_{eV}$ with maximal accuracy. In the rest of this section, we describe these three steps in more detail.

For the first contribution (i), following Hössjer et al. (2016) we divide expected squared allele frequency change between the two time points at which genetic data is collected into two components *I* and *II*, and study conditions under which the impact of *II* is negligible. In order to motivate more closely this first aspect of our article, we will start by explaining the meaning of these two terms *I* and *II*.

The first component *I* was analyzed by Whitlock and Barton (1997) and Hössjer et al. (2014), and it quantifies how much standardized covariances of allele frequency change increase or how much the gene diversity decreases between the two time points at which genetic data is collected. We will refer to *I* as the drift term, since it is mainly genetic drift that causes loss of genetic variation, and for this reason *I* is usually the most important source of genetic change. Indeed, gene diversity decrease *I* is equivalent to gene identity increase, a haploid approximation of increased inbreeding that is of major concern for short term protection of species (Franklin 1980; Jamieson and Allendorf 2012). For this reason the gene diversity effective size $N_{eGD}$ is of interest for conservation since it only involves the genetic drift term *I* but not the other term *II*. $N_{eGD}$ is however of more relevance for long term than for short term conservation since it approximates the additive genetic variance effective size $N_{eAV}$ (Franklin 1980; Hössjer et al. 2016). This is important since the frequently used conservation guideline for long term survival, that stipulates that $N_e$ should be larger than 500 (Franklin 1980; Jamieson and Allendorf 2012) or larger than 1000 (Frankham et al. 2014; Pérez-Pereira et al. 2022), relates to $N_{eAV}$ (Ryman et al. 2019). However, since $N_{eGD}$ (and $N_{eAV}$) is difficult to estimate in practice, it is important to assess how well it is approximated by $N_{eV}$.

The second term *II* was introduced in Hössjer et al. (2016) and it quantifies how much allele frequency change in the past, before the first time point when data is collected, is correlated with allele frequency change between the two time points of data collection, with a negative correlation corresponding to a positive value of *II*. We could therefore refer to $-II$ as a correlation between allele frequency change of the past and the present. But since *II* vanishes when all subpopulations are isolated and additionally the same subpopulation weights are used at the two time points at which genetic data is collected, it follows that *II* is mainly caused by migration. For this reason we will speak of *II* as a migration or gene flow term. It is also the case that *II* is present only when there is subpopulation differentiation at the first time point of data collection.

In order to shed further light on the relation between $N_{eGD}$ and $N_{eV}$, we continue the analysis of Hössjer et al. (2016) and express genetic drift *I* and gene flow *II* in terms of how subpopulations are weighted, and also in terms of parameters of the population genetic model such as the local census and effective sizes and the migration rates between subpopulations. In particular, we demonstrate that *II* is highly dependent on local census sizes, whereas *I* is virtually independent of them (although local census sizes were introduced in Hössjer et al. (2014), they had little impact on $N_{eV}$ since *II* was not included as a component of allele frequency change in that article).

It is of particular interest to find conditions under which it is possible to estimate $N_{eV}$ in such a way that *II* is eliminated. It was shown in Hössjer et al. (2016) that the gene flow contribution *II* to the variance effective size vanishes when subpopulations are weighted proportionally to their long term reproductive contribution (Hill 1972; Nagylaki 1980; Whitlock and Barton 1997). This means that each subpopulation receives a weight that corresponds to the fraction of ancestors, many generations ago, that originated from this particular subpopulation. When subpopulations are weighted in this way, the overall frequency of an allele in the metapopulation changes over time in such a way that only genetic drift (term *I*) contributes, whereas the effects of migration into different subpopulations cancel out ($II=0$). These so called reproductive subpopulation weights give rise to a version of the variance effective size that we refer to as $N_{eVMeta}$. It turns out that $N_{eVMeta}$ is of particular interest for long term conservation, since under migration–drift equilibrium $N_{eVMeta}$ not only equals $N_{eGD}$ and $N_{eAV}$, but also the eigenvalue effective size $N_{eE}$ (Ewens 1982, 2004), which is known to reflect the long term genetic behavior of a population.

In spite of the relevance of $N_{eVMeta}$ for conservation, it is a challenge to use this effective size in practice since its subpopulation weights involve migration rates between subpopulations, which are difficult to estimate. It is possible, though, to find simplified expressions for *I* and *II* under migration–drift equilibrium, using perturbation theory and eigenvalue decomposition of matrices (Horn and Johnson 1985; Friswell 1996; Van der Aa et al. 2007). Although perturbation results for eigenvalues have previously been applied to population genetics (Maruyama 1970a; Nagylaki 1980, 1995; Hössjer 2015) it seems that our perturbation results for eigenvectors are new. Based on this analysis we demonstrate, for some particular models, that it is possible to eliminate the impact *II* of migration by maximizing the variance effective size with respect to subpopulation weights, so that the corresponding $N_{eV}$ approximates $N_{eVMeta}$.

For the second contribution (ii) of this article, we demonstrate that when the impact *II* of gene flow is not eliminated, under certain conditions it elevates allele frequency change over long time intervals to such an extent that the traditional (forward) version of $N_{eV}$ is undefined. For this reason we define two novel notions of variance effective size, the intermediate and backward versions of $N_{eV}$. The intermediate version corresponds to a frequently used estimator of variance effective size due to Jorde and Ryman (2007), and although it is more stable than the forward version of $N_{eV}$, it shares the drawback of sometimes being undefined for long time intervals of genetic change. The backward version of $N_{eV}$, on the other hand, exists for most populations, and it is also the version of variance effective size that most closely relates to $N_{eGD}$ and $N_{eE}$, since it lessens the impact of the gene flow term *II* more than the other two versions of variance effective size. We demonstrate, numerically and analytically, that the forward, intermediate and backward versions of the local variance effective size are very close for large subdivided populations, unless the time interval is very long. On the other hand, the three effective sizes differ substantially for a small and subdivided population, and moderate or large time intervals.

For the third contribution (iii) of this article, we give explicit expressions for the length of the time interval that maximizes the accuracy of estimates of $N_{eGD}$ and all three versions of $N_{eV}$, for any type of subpopulation weights. This optimal length is proportional to the eigenvalue effective size $N_{eE}$, with a constant of proportionality that depends on characteristics of the population as well as the type of effective size being used, including subpopulation weights. This reinforces that the three variance effective sizes behave differently for small subdivided populations (when $N_{eE}$ is small).

Our paper is organized as follows: We start by defining the population genetic model in Sect. 2, and the framework of genetic variation in terms of one single biallelic marker in Sect. 3. This makes it possible in Sect. 4 to introduce the matrix analytic framework for how covariances of allele frequency change, gene diversities and fixation indeces evolve over time. The various notions of effective size are introduced in Sect. 5, migration–drift equilibrium is the topic of Sect. 6, the impact of the length of the time interval on effective size is analyzed in Sect. 7, and the optimal time interval in terms of accurately estimating effective size is studied in Sect. 8. Then analysis of a real data set in Sect. 9 and a discussion in Sect. 10 concludes. A summary of the most important notation is provided in Table 1, whereas some of the numerical results and all proofs are collected in the appendices.

**Table 1:** A summary of the most important notation used in this article

Quantity	Description
*s*	Number of subpopulations
*x*, *y*	Index of a subpopulation ($\in \{1,\ldots ,s\}$)
*t*	Time index, in units of generations ($\in \{-T,-T+1,\ldots \}$)
*T*	Number of generations ago ($t=-T$) when the founder population lived
$\tau$	Length of time interval along which genic change is assessed
$B_{xy}$	Backward migration rate from *x* to *y* or the fraction of parents of individuals in subpopulation *x* that originate from subpopulation *y* one generation ago
$\varvec{B}$	Square matrix $(B_{xy})_{x,y=1}^s$ of order *s* with all backward migration rates
$m^\prime$	Migration rate of island model ($B_{xy}=m^\prime /(s-1)$ when $x\ne y$)
*m*	Fraction of parents in the island model that originate from the whole population one generation ago ($=sm^\prime /(s-1)$)
$w_x$	Weight of subpopulation *x* at the start ($=t$) of the time interval along which genetic change is assessed
$\varvec{w}$	Vector of subpopulation weights ($=(w_1,\ldots ,w_s)$) at the start of the time interval along which genetic change is assessed
$v_x$	Weight of subpopulation *x* at the end ($=t+\tau$) of the time interval along which genetic change is assessed
$\varvec{v}$	Vector of subpopulation weights ($=(v_1,\ldots ,v_s)$) at the end of the time interval along which genetic change is assessed
$\varvec{e}_x$	Vector of local subpopulation weights ($=(0,\ldots ,0,1,0,\ldots ,0)$) that only assigns a positive weight to subpopulation *x* (a 1 in position *x*)
$\varvec{\gamma }$	Vector of reproductive subpopulation weights ($=(\gamma _1,\ldots ,\gamma _s)$). $\gamma _x$ is the fraction of ancestors originating from subpopulation *x* many generations ago
*A*	One of the two alleles of a biallelic marker
*p*	Frequency of *A* in all subpopulations in the founder generation
$p_{tx}$	Frequency of allele *A* in subpopulation *x* and generation *t*
$p_t$	Weighted frequency of allele *A* in the whole population in generation *t*, when subpopulations are weighted as $\varvec{w}$ ($=\sum _x w_xp_{tx}$)
$p_{t+\tau }$	Weighted frequency of allele *A* in the whole population in generation $t+\tau$, when subpopulations are weighted as $\varvec{v}$ ($=\sum _x v_xp_{t+\tau ,x}$)
$\varvec{1}_n$	Column vector of length *n* with ones in all positions
$h_{tx}$	$1-h_{tx}$ is the standardized covariance of allele frequency change from the base generation to generation *t*, between subpopulations *x* and *y*
$\varvec{h}_t$	Column vector of length $s^2$ with all the one minus standardized covariances at time *t* ($=(h_{txy})_{x,y=1}^s$)
$H_{txy}$	Gene diversity between subpopulations *x* and *y* in generation *t*
$\varvec{H}_t$	Column vector of length $s^2$ with all gene diversities at time *t* ($=(H_{txy})_{x,y=1}^s$)
$\varvec{A}$	Square matrix of order $s^2$ describing a linear time recursion of $\varvec{h}_t$ and $\varvec{H}_t$
$\lambda$	Largest eigenvalue of $\varvec{A}$
$\varvec{r}$	Right eigenvector of $\varvec{A}$ with eigenvalue $\lambda$
$F_{ST,t}$	Fixation index in generation *t*
$F_{ST}^{\textrm{eq}}$	Fixation index under migration–drift equilibrium
$N_e$	Generic notation for effective population size
$N_{cx}$	Local census size of subpopulation *x* ($=N_c$ if all $N_{cx}$ are identical)
$N_{ex}$	Local effective size of an isolated subpopulation *x* ($=N_e$ if all $N_{ex}$ are identical)
*Q*	Quantity used for defining type of effective population size
$N_{eQ}$	Generic notation for effective population size of type *Q*
$N_{eQ}^{\textrm{eq}}$	Generic notation for effective size of type *Q* at migration–drift equilibrium
$N_{eQ\varvec{w}\varvec{v}}$	Effective population size when subpopulations are weighted as $\varvec{w}$ and $\varvec{v}$ at the two end points of the interval along which genetic change is assessed
$N_{eQ\varvec{w}}$	Effective population size when subpopulations are weighted as $\varvec{w}$ at both end points of the interval along which genetic change is assessed ($=N_{eQ\varvec{w}\varvec{w}}$)
$N_{eQMeta}$	Effective size of type *Q* for the metapopulation ($=N_{eQ\varvec{\gamma }}=N_{eQ\varvec{\gamma }\varvec{\gamma }}$)
$N_{eQRx}$	Realized local effective size of type *Q* for subpopulation *x* ($=N_{eQ\varvec{e}_x}=N_{eQ\varvec{e}_x\varvec{e}_x}$). It equals $N_{ex}$ when *x* is isolated from the other subpopulations
$N_{eQRxy}$	Realized local effective size of type *Q* when subpopulations *x* and *y* ($x\ne y$) receive full weight at the two end points of the time interval ($=N_{eQ\varvec{e}_x\varvec{e}_y}$)
$N_{eGD}$	Generic notation for gene diversity effective size
$N_{eAV}$	Generic notation for additive genetic variance effective size
$N_{eV}$	Generic notation for forward version of variance effective size
$N_{eV}^{\textrm{int}}$	Generic notation for intermediate version of variance effective size
$N_{eV}^{\textrm{back}}$	Generic notation for backward version of variance effective size
$N_{eE}$	Eigenvalue effective size (it has only one version that describes long term change at migration–drift equilibrium and it does not involve subpopulation weights)
$I_{T+t}(\tau )$	Contribution to expected squared allele frequency change between generations *t* and $t+\tau$ from decreased gene diversity between these two generations. $I_{T+t}(\tau )$ is referred to as a genetic drift term (generic notation *I*, equals $I_\infty (\tau )$ at migration–drift equilibrium)
$II_{T+t}(\tau )$	$-II_{T+t}(\tau )$ is the contribution, to expected squared allele frequency change between generations *t* and $t+\tau$, from correlation between allele frequency change between these two generations and allele frequency of the past, before time *t*. $II_{T+t}(\tau )$ is mainly caused by migration and it is therefore often referred to as a migration or gene flow term (generic notation *II*, equals $II_\infty (\tau )$ at migration–drift equilibrium)

## Population Genetic Model

We will study the genetic composition of a structured (or subdivided) population that evolves over time in terms of non-overlapping generations $t=-T,-T+1,\ldots$, where $-T\le 0$ is a founder generation. The population has *s* subpopulations $x=1,\ldots ,s$, whose local census sizes $N_{cx}$ and local effective sizes $N_{ex}$ under isolation do not change over time. The subpopulations are not isolated, but rather connected though gene flow, as summarized by an irreducible backward migration matrix $\varvec{B}=(B_{xy})$ of order *s*, where $B_{xy}$ is the expected fraction of gene copies in *x* that in the previous generation migrated from *y*.

The most well known type of subdivided population is the island model (Wright 1943; Maruyama 1970b), for which $N_{ex}=N_e$ and $N_{cx}=N_c$ are the same for all subpopulations. The migration rates $B_{xy}=m^{\prime }/(s-1) = m/s$ between all pairs $x\ne y$ of subpopulations are the same as well, so that in each generation $m^{\prime }={1-B}_{xx}$ is the fraction of offspring of subpopulation *x* whose parents migrated from any other subpopulation $\{y; \, y\ne x\}$. On the other hand, *m* can be thought of as the fraction of offspring of *x* whose parents originate from a global gene pool, with equal contribution from all subpopulations (including *x* itself). The one- and two-dimensional stepping stone models (Kimura 1953; Kimura and Weiss 1964; Weiss and Kimura 1965; Durrett 2008) correspond to a subdivided population where migration from *y* to *x* ($B_{xy}>0$) is possible only when these two subpopulations are neighbors.

It is assumed that the population reproduces in such a way that migration precedes fertilization. More specifically, reproduction between generations *t* and $t+1$ involves the following three steps:

1. (Gamete formation) Within each subpopulation *x* of generation *t* an infinitely large pre-migration gene pool is constructed as follows: $2N_{ex}$ gene copies (corresponding to $N_{ex}$ diploid breeders) are drawn without replacement from all $2N_{cx}$ gene copies (corresponding to $N_{cx}$ diploid individuals) of this subpopulation *x* at time *t*. All $2N_{ex}$ drawn gene copies multiply and contribute in equal proportions $1/(2N_{ex})$ to the infinite pre-migration pool of *x*. These *s* pre-migration gene pools ($x=1,\ldots ,s$) are constructed independently for all subpopulations, without any exchange of genetic material.
2. (Migration) The *s* pre-migration gene pools of step 1 mix, so that *s* post-migration pools are formed. In particular, the post-migration pool of subpopulation *x* is a mixture of the pre-migration pools of subpopulations $1,\ldots ,s$ in proportions $B_{x1},\ldots ,B_{xs}$.
3. (Fertilization) The $2N_{cx}$ gene copies (corresponding to $N_{cx}$ diploid individuals) of subpopulation *x* and generation $t+1$ are formed by sampling $2N_{cx}$ genes from the post-migration gene pool of *x*. This is done independently between all subpopulations $x=1,\ldots ,s$.

We refer to this reproduction scenario as MF/FF, an acronym for migration preceding fertilization, with fixed migrant proportions and fixed migrant allele frequencies. It was used in Hössjer et al. (2013) for the island model and in Hössjer et al. (2014) and Olsson et al. (2017) for subdivided populations of general form. A number of other, closely related reproduction schemes were studied in the context of the island model by Hössjer et al. (2013) and more generally by Hössjer and Ryman (2014).

## Genetic Variation at a Biallelic Marker

Our main focus is to study how the genetic composition of the population of Sect. 2 changes between two time points *t* and $t+\tau$, where $\tau$ is a positive integer. Typically genetic data from many biallelic markers are used to represent the genetic composition at time *t* and $t+\tau$. For our theoretical investigations in Sects. 3–8, it will be sufficient though to study one single biallelic marker, as a representative of any of the markers that are part of the data set. For this reason we consider a marker with alleles *A* and *a* and let $p_t$ be the frequency of allele *A* in generation *t*. For a subdivided population we need to keep track of the frequency $p_{tx}$ of *A* in all subpopulations *x* at each time point *t*. In order to obtain one single allele frequency at time *t* and $t+\tau$, we will weight subpopulations as $\varvec{w}=(w_1,\ldots ,w_s)$ and $\varvec{v}=(v_1,\ldots ,v_s)$ at these two time points, where $w_x$ and $v_x$ are non-negative numbers satisfying $\sum _x w_x=\sum _x v_x=1$. The accompanying subpopulation weighted frequencies of *A*, at time *t* and $t+\tau$, are

1 $$\begin{aligned} \begin{array}{rcl} p_t &{}=&{} \sum _x w_x p_{tx},\\ p_{t+\tau } &{}=&{} \sum _x v_xp_{t+\tau ,x}. \end{array} \end{aligned}$$

The subpopulation weights in (1) play a crucial role in this paper. They may for instance reflect the sampling scheme of time points *t* and $t+\tau$, although this is not necessary. Local subpopulation weights at time *t* correspond to giving some subpopulation *x* full weight ($w_x=1$), whereas none of the other subpopulation contribute to $p_t$ ($w_y=0$ for any $y\ne x$). With vector notation this is phrased as $\varvec{w}=\varvec{e}_x$, where $\varvec{e}_x=(0,\ldots ,0,1,0,\ldots ,0)$ has a one in position *x* and zeros elsewhere. Similarly, $v_x=1$ if subpopulation weight *x* receives full weight at time $t+\tau$, or equivalently $\varvec{v}=\varvec{e}_x$. Global subpopulation weights at time *t* and $t+\tau$ assign positive values $w_x>0$ and $v_x>0$ respectively, to all subpopulations *x*. When the long term evolution of the population is of interest, it is appropriate to use reproductive subpopulation weights $\varvec{w}=\varvec{v}=\varvec{\gamma }=(\gamma _1,\ldots ,\gamma _s)$ at both time points, since a fraction $\gamma _x$ of all gene copies originated from subpopulation *x* many generations ago (Nagylaki 1980, 2000; Hössjer and Ryman 2014). This weight vector $\varvec{\gamma }$ is the equilibrium distribution of a Markov chain with state space $\{1,\ldots ,s\}$ and transition matrix $\varvec{B}$, and it corresponds to a probability distribution for the subpopulation ancestry of a gene copy. Assuming that $\varvec{B}$ is irreducible, $\varvec{\gamma }$ is the unique probability vector satisfying $\varvec{\gamma }=\varvec{\gamma }\varvec{B}$, with $\varvec{\gamma }=(1,\ldots ,1)/s$ for the island model. Consequently, $\gamma _x$ quantifies the long term contribution of *x* to the metapopulation, or as mentioned above, $\gamma _x$ is the fraction of ancestors that originated from *x*, many generations back in time.

## Standardized Covariances, Gene Diversities, and Fixation Indeces

In this section we define a number of concepts needed in Sect. 5 when various types of effective size are introduced. Following Hössjer et al. (2016), assume that all subpopulations have the same frequency $p_{-T,x}=p$ of allele *A* at the founder generation at $t=-T$. This is no essential restriction, since we will mainly consider equilibrium conditions when $T\rightarrow \infty$.

### Standardized Covariances

The standardized covariance between a pair *x*, *y* of subpopulations at time point $t\in \{-T,-T+1,\ldots \}$, is defined as

2 $$\begin{aligned} f_{txy} = \frac{\text{ Cov }(p_{tx}-p,p_{ty}-p)}{p(1-p)} = 1 - h_{txy}. \end{aligned}$$

Equivalently, $f_{txy}$ is the correlation coefficient between the alleles of two gene copies drawn independently from subpopulations *x* and *y* at time *t* (with replacement if $x=y$), see for instance Cockerham (1969). It was shown in Hössjer et al. (2014) that the column vector $\varvec{h}_t = (h_{txy})$ of length $s^2$ satisfies a recursive relation

3 $$\begin{aligned} \varvec{h}_{t+1} = \varvec{A}\varvec{h}_t, \end{aligned}$$

where $\varvec{A}=(A_{xy,zu})$ is a square matrix of order $s^2$ with elements

4 $$\begin{aligned} A_{xy,zu} = \left( 1-\frac{1}{2N_{cx}}\right) ^{1(x=y)} B_{xz}B_{yu} \left( \frac{1-\frac{1}{2N_{ez}}}{1-\frac{1}{2N_{cz}}} \right) ^{1(z=u)}. \end{aligned}$$

Similar types of recursions were originally developed by Malécot (1951), see also Whitlock and Barton (1997). Since all standardized covariances vanish at the founder generation, it follows that

5 $$\begin{aligned} \varvec{h}_{-T}=\varvec{1}_{s^2} = (1,\ldots ,1)^T \end{aligned}$$

is a vector of $s^2$ ones. Notice that the initial condition (5) and the linear recursion (3) determine the value of $h_{txy}$ for all *t*, *x*, *y*.

### Gene Diversities

The gene identity (gene diversity) $F_{txy}$ ($H_{txy}$) between a pair of subpopulations at time *t* is the probability that two randomly chosen gene copies of subpopulations *x* and *y*, drawn with replacement if $x=y$, have the same (different) alleles. It turns out that the time recursive behavior of gene diversities is very similar to that of standardized covariances. In order to motivate this we notice that allele frequencies at time $t>-T$ are unknown from the perspective of the base generation $-T$, and therefore

$$\begin{aligned} H_{txy} = E\left[ p_{tx}(1-p_{ty}) + (1-p_{tx})p_{ty}\right] . \end{aligned}$$

This implies that the gene diversities at time *t* and $t+\tau$ are given by

6 $$\begin{aligned} \begin{array}{rcl} H_t &{}=&{} E\left[ 2p_t(1-p_t)\right] = \sum _{x,y} w_xw_y H_{txy},\\ H_{t+\tau } &{}=&{} E\left[ 2p_{t+\tau }(1-p_{t+\tau })\right] = \sum _{x,y} v_x v_y H_{t+\tau ,xy}. \end{array} \end{aligned}$$

By this we mean that $H_t$ is the probability that two gene copies, drawn randomly with replacement from the population at time *t*, have different alleles, given that $w_x$ is the probability of drawing each gene from *x*. Likewise, $H_{t+\tau }$ is the probability that two randomly drawn gene copies at time $t+\tau$ have different alleles, if subpopulations are chosen with probabilities $v_x$. It was shown in Hössjer et al. (2014) that the column vector $\varvec{H}_t = (H_{txy})$ of length $s^2$ satisfies the same recursion as in (3), i.e.

7 $$\begin{aligned} \varvec{H}_{t+1} = \varvec{A}\varvec{H}_t, \end{aligned}$$

for $t=-T,-T+1,\ldots$. Since $p_{-T,x}=p$ by assumption, we have that $H_{-Txy}=2p(1-p)$ for all *x*, *y*, and consequently

8 $$\begin{aligned} \varvec{H}_{-T} = 2p(1-p)\varvec{1}_{s^2}. \end{aligned}$$

Comparing (3) and (5) with (7) and (8), we find that the numbers $h_{txy}$ obtained from the standardized covariances are equivalent to the gene diversities $H_{txy}$, up to a multiplicative constant, i.e.

9 $$\begin{aligned} H_{txy} = 2p(1-p)h_{txy} \end{aligned}$$

for all *t*, *x*, *y*.

A concept closely related to (6) is the collection of gene diversities *without replacement*. They are defined as

10 $$\begin{aligned} \begin{array}{rcl} \tilde{H}_t &{}=&{} \sum _{x,y} w_xw_y \tilde{H}_{txy},\\ \tilde{H}_{t+\tau } &{}=&{} \sum _{x,y} v_x v_y \tilde{H}_{t+\tau ,xy}, \end{array} \end{aligned}$$

the probabilities that two gene copies, drawn randomly *without* replacement at the same time point *t* and $t+\tau$ respectively, have different alleles. Likewise, $\tilde{H}_{txy}$ is the probability that two gene copies, drawn at randomly without replacement from *x* and *y* at time *t*, have different alleles. It was shown in Hössjer et al. (2014) that the column vector $\tilde{\varvec{H}}_t=(\tilde{H}_{txy})$ of gene diversities without replacement satisfies a recursion

11 $$\begin{aligned} \tilde{\varvec{H}}_{t+1} = \varvec{D}\tilde{\varvec{H}}_t \end{aligned}$$

for $t=-T,-T+1,\ldots$, where $\varvec{D}= (D_{xy,zu})$ is a square matrix of order $s^2$ with elements

12 $$\begin{aligned} D_{xy,zu} = B_{xz}B_{yu} \left( 1- \frac{1}{2N_{ez}}\right) ^{1(z=u)}. \end{aligned}$$

Note that the definitions of $\tilde{H}_{txy}$ and $H_{txy}$ are the same when $x\ne y$. Moreover, since $(2N_{cx}-1)/(2N_{cx})$ is the probability that two gene copies, drawn randomly with replacement from *x*, are different copies, it follows that

13 $$\begin{aligned} \tilde{H}_{txy} = H_{txy} \left( \frac{2N_{cx}}{2N_{cx}-1}\right) ^{I(x=y)} \end{aligned}$$

for all *x*, *y*. In particular, a comparison between (8) and (13) reveals that $\tilde{\varvec{H}}_{-T}$ is virtually independent of the census sizes $N_{cx}$ when these are large. Since the elements (12) of the linear recursion matrix $\varvec{D}$ do not involve any census sizes, it follows that $\tilde{H}_{txy}$ are virtually independent of census sizes as well, for any *t*, *x*, *y*. Making use of (13) again, we conclude that the gene diversities $H_{txy}$ are virtually independent of the local census sizes as well.

### Fixation Index

The most well known measure of genetic differences between subpopulations is the fixation index $F_{ST}$ (Malécot 1948, Wright 1949; Weir and Cockerham 1984; Bhatia et al. 2013). Here we will use a version of the fixation index referred to as the coefficient of gene differentiation by Nei (1973) and subsequently generalized to multiallelic loci in Nei (1977). The fixation index is conveniently defined in terms of allele frequency differences between subpopulations, and we will study $F_{ST}$ in each generation *t* from the perspective of the base generation $-T$, so that allele frequencies at $t>-T$ are unknown. Following the argument in Hössjer et al. (2016), the fixation index at time *t* is then predicted by

14 $$\begin{aligned} F_{ST,t} = \frac{\sum _{x=1}^s w_x E\big [(p_{tx}-p_t)^2\big ]}{E[p_t(1-p_t)]} = \frac{\sum _{x,y} w_x w_y h_{txy} - \sum _x w_x h_{txx}}{\sum _{x,y} w_x w_y h_{txy}}, \end{aligned}$$

where in the last step of (14) we first divided the numerator and denominator by $p(1-p)$, and then invoked the definition of $h_{txy}=1-f_{txy}$ in (2). In order for the fixation index to be nonzero, it is required that at least two subpopulation weights $w_x$ are nonzero. We will mainly use (14) in the context of reproductive population weights $\varvec{w}=\varvec{\gamma }$.

## Effective Sizes

The idea of effective size is to find a simple population that serves as a yardstick and shares some properties with the structured population of interest. The Wright–Fisher population (WF) is usually used for this purpose. It is a special case of the model of Sect. 2 that corresponds to a homogeneous population ($s=1$) with equal census and effective size ($N_{c1}=N_{e1}=N$). An effective size of type *Q* (notated as $N_{eQ}$) is the size of a WF population that exhibits the same value of a certain quantity *Q* as the given structured population. Typically *Q* quantifies how fast the genetic composition of the population changes between time points *t* and $t+\tau$, and we will assume that it takes the value $(1-1/(2N))^\tau$ for a WF population of size *N*, so that

15 $$\begin{aligned} Q = Q(\tau ) = 1 - \left( 1 - \frac{1}{2N_{eQ}}\right) ^\tau . \end{aligned}$$

Solving for the effective size in (15) we find that

16 $$\begin{aligned} N_{eQ} = \frac{1}{2 \{1-[1-Q(\tau )]^{1/\tau }\}}. \end{aligned}$$

Equation (16) is very close to a formula for the effective size that appears at the bottom of Page 525 of Luikart et al. (1999). Since they have $2N_{eQ}+1$ rather than $2N_{eQ}$ in the denominator of (15), they end up with an additional term $-0.5$ in the expression for $N_{eQ}$, and they also include extra terms that correct for estimation bias of $Q(\tau )$ due to having finite samples of genetic data at time points *t* and $t+\tau$.

When $N_{eQ}\gg \tau$, the right hand side of (15) is well approximated by a first order Taylor expansion of $g(x)=1-(1-x)^\tau \approx \tau x$ around $x=0$. This gives rise to the simpler and approximate definition

17 $$\begin{aligned} N_{eQ,\textrm{add}} = \frac{\tau }{2Q(\tau )}. \end{aligned}$$

Sometimes (17) is referred to as the additive approach (Waples 1989; Luikart et al. 1999), as opposed to the exact multiplicative approach (15). Although the additive approximation often works well, it can sometimes be inaccurate when $\tau$ gets large, in particular for populations that experience bottlenecks (Richards and Leberg 1996; Luikart et al. 1999). Another important difference between the multiplicative and additive approaches is that $N_{eQ,\textrm{add}}$ always exists, as long as *Q* is positive, whereas in order for $N_{eQ}$ to have a finite positive value we must require $0<Q<1$. Although $Q<1$ is guaranteed for a Wright–Fisher population, for a subdivided population in general *Q* may sometimes exceed in 1.

In this paper we will mainly focus on loss of gene diversity ($Q=GD$) and variance of allele frequency change ($Q=V$). But we will also consider the eigenvalue effective size, for which $Q=E$ corresponds to the largest eigenvalue of a certain matrix. This effective size does not follow the general pattern (15) and (16) of genetic change between two time points *t* and $t+\tau$, but rather it quantifies the long term loss of genetic diversity at migration–drift equilibrium.

### Notation for Local and Global Effective Sizes

We will assume that subpopulations are weighted as $\varvec{w}$ and $\varvec{v}$ at time *t* and $t+\tau$, and in order to highlight the impact of these subpopulation weights we sometimes write $N_{eQ}=N_{eQ\varvec{w}\varvec{v}}$, and in particular $N_{eQ}=N_{eQ\varvec{w}}$ when the same weight vector $\varvec{w}=\varvec{v}$ is used at both time points. For an effective size of type $Q\in \{GD,V\}$ we also adopt the notation of Laikre et al. (2016) and write $N_{eQMeta}=N_{eQ\varvec{\gamma }}$ for the effective population size of the metapopulation. This corresponds to using reproductive weights at both time points ($\varvec{w}=\varvec{v}=\varvec{\gamma }$). The quantity $N_{eQRx}=N_{eQ\varvec{e}_x}$ refers to the realized local effective size of subpopulation *x*, and it corresponds to using the same local weight vector at both time points ($\varvec{w}=\varvec{v}=\varvec{e}_x$). The term realized was introduced in Laikre et al. (2016) and Ryman et al. (2019) to emphasize the fact that due to migration $N_{eQRx}$ typically differs from $N_{ex}$, although the two quantities are identical when *x* is isolated from the other subpopulations. When two different subpopulations *x* and *y* receive full weight at time points *t* and $t+\tau$, i.e. $\varvec{w}=\varvec{e}_x$, $\varvec{v}=\varvec{e}_y$, and $x\ne y$, we write $N_{eQRxy}=N_{eQ\varvec{e}_x\varvec{e}_y}$ for the corresponding realized effective size.

The eigenvalue effective size $N_{eE}$, on the other hand, is a property of the metapopulation, and therefore it does not involve subpopulation weights.

### Gene Diversity Effective Size

The gene diversity effective size $N_{eGD}$ between the two time points *t* and $t+\tau$ is defined as the size of an ideal Wright–Fisher population that exhibits the same relative gene diversity decline (locally or globally for the metapopulation), during this time interval, as for the studied structured population. In mathematical terms, this corresponds to the quantity

18 $$\begin{aligned} Q(\tau ) = 1 - \left( 1 - \frac{1}{2N_{eGD}}\right) ^\tau = \frac{H_t - H_{t+\tau }}{H_t} =: I, \end{aligned}$$

where $I=I_{T+t}(\tau )$, the relative decline of gene diversity, quantifies how much genetic drift there has been between generations *t* and $t+\tau$.

The effective size in (18) was referred as a haploid inbreeding effective size with replacement in Hössjer et al. (2014), since gene diversity decrease is equivalent to gene identity increase, a haploid analogue of increased inbreeding. It was shown in Hössjer et al. (2016) that $N_{eGD}$ is a good approximation of the additive genetic variance effective size $N_{eAV}$, which is of interest for long term conservation of species. Recall from the discussion at the end of Sect. 4.2 that all $H_{txy}$ and $H_{t+\tau ,xy}$ are essentially independent of the local census sizes of all subpopulations. From this it follows that $H_t$, $H_{t+\tau }$, *I*, and $N_{eGD}$ are functions of the migration rates in $\varvec{B}$ and the local effective sizes $N_{ex}$, whereas they are essentially independent of the local census sizes $N_{cx}$. Since *I* is nonzero even when all subpopulations are isolated, the contribution of all $N_{ex}$ is most fundamental to *I*, and for this reason we will refer to it as a genetic drift term.

Since the gene diversities $H_t$ and $H_{t+\tau }$ are non-negative, it follows that the term *I* does not exceed unity ($I\le 1$). It may happen though that *I* is negative when local subpopulations weights of *x* are used at both time points ($\varvec{w}= \varvec{v}= \varvec{e}_x$) and migration into *x* causes the gene diversity to increase ($H_{t+\tau }>H_t$). Then formally $N_{eGD}=\infty$.

### Variance Effective Size

#### Forward Approach

The variance effective size $N_{eV}$ is the size of an ideal and spatially homogeneous population whose standardized variance of allele frequency change between time points *t* and $t+\tau$ is the same as in the studied structured population, see for instance Sect. 7.6.3 of Crow and Kimura (1970). It is instructive to first introduce $N_{eV}$ for a population that is either spatially homogeneous ($s=1$) or has a substructure that is ignored. The traditional definition

19 $$\begin{aligned} 1 - \left( 1-\frac{1}{2N_{eV}^{\textrm{trad}}}\right) ^\tau = \frac{\text{ Var }(p_{t+\tau }-p_t|p_t)}{p_t(1-p_t)} = F_{\textrm{trad}} \end{aligned}$$

quantifies variance of allele frequency change conditionally on allele frequencies of generation *t*. If mutations and selection of a homogeneous population is ignored, then typically allele frequency change of the past (before time *t*) is uncorrelated with allele frequency change of the present (between time points *t* and $t+\tau$). This implies that $E(p_{t+\tau }|p_t)=p_t$, so that the variance in (19) equals $E[(p_{t+\tau }-p_t)^2|p_t]$. It turns out that the latter quantity is preferable to use in more general settings (such as a subdivided population) when possibly $E(p_{t+\tau }|p_t)\ne p_t$, due to the fact that allele frequency change of the past might be correlated with allele frequency change between time points *t* and $t+\tau$. We therefore define the variance effective size of a subdivided population (with subpopulation weights $\varvec{w}$ and $\varvec{v}$) as

20 $$\begin{aligned} Q(\tau ) = 1 - \left( 1-\frac{1}{2N_{eV}}\right) ^\tau = \frac{E[(p_{t+\tau }-p_t)^2]}{E[p_t(1-p_t)]} = F. \end{aligned}$$

Equation (20) differs from (19) in that the numerator and denominator of the genetic drift term *F* are averaged with respect to $p_t$. Indeed, it is well known (Ewens 1982; Hössjer and Ryman 2014; Hössjer et al. 2014, 2016) that typically $E[(p_{t+\tau }-p_t)^2|p_t]/[p_t(1-p_t)]$ is not a fixed number for a structured population, but rather a function of $p_t$. This makes the more general definition of genetic drift in (20) preferable for a metapopulation with subpopulations, since the impact of $p_t$ is averaged out. We will refer to (20) as the *forward* definition of $N_{eV}$, since allele frequency change is normalized, in the denominator, as a function of allele frequencies at time *t*, the left end point of the interval $[t,t+\tau ]$, and from the perspective of this time point the allele frequency change in the numerator of (20) takes place forwards in time. Note that the traditional definition (19) of variance effective size is based on the forward approach as well, and it can be seen that (20) is a generalization of (19). In particular, when $\tau =1$ and the population is homogeneous, both of (19) and (20) reduce to the well know formula $N_{eV} = p_t(1-p_t)/[2\text{ Var }(p_{t+1}-p_t|p_t)]$, see for instance Crow and Kimura (1970, Eq. 7.6.3.25).

Following Hössjer et al. (2016), where the special case $\varvec{w}=\varvec{v}$ was treated, we rewrite the right hand side of (20) as

21 $$\begin{aligned} \begin{array}{lll} 1 - \left( 1-\frac{1}{2N_{eV}}\right) ^\tau &=&\frac{E[(p_{t+\tau }-p_t)^2]}{E[p_t(1-p_t)]}\\ &&= \frac{E[(p_{t+\tau }-p)^2]-E[(p_t-p)^2]}{E[p_t(1-p_t)]} + \frac{-2\textrm{Cov}(p_{t+\tau }-p_t,p_t-p)}{E[p_t(1-p_t)]}\\ &&= \frac{E[(p_{t+\tau }-p)^2]/[p(1-p)]-E[(p_t-p)^2]/[p(1-p)]}{E[p_t(1-p_t)]/[p(1-p)]}\\ &&\quad + \frac{-2\textrm{Cov}(p_{t+\tau }-p_t,p_t-p)/[p(1-p)]}{E[p_t(1-p_t)]/[p(1-p)]}\\ &&= I + II. \end{array} \end{aligned}$$

The first term *I* on the right hand side of (21) is identical to the genetic drift term *I* that appears in the definition (18) of the gene diversity effective size. Indeed, it follows from (2) and (21) that

22 $$\begin{aligned} I = I_{T+t}(\tau ) = \frac{\sum _{x,y} w_xw_y h_{txy}-\sum _{x,y} v_xv_y h_{t+\tau ,xy}}{\sum _{x,y} w_xw_y h_{txy}} \end{aligned}$$

can be expressed in terms of $h_{txy}$ and $h_{t+\tau ,xy}$ for all pairs *x*, *y* of subpopulations, which in view of (9) are proportional to the corresponding gene diversities that appear in the genetic drift term *I* of (18).

The second term *II* of (21) is only present in a subdivided population, and therefore it follows from (18) and (21) that $N_{GD}=N_{eV}$ for homogeneous populations. For a subdivided population, $-II$ accounts for the correlation between allele frequency change up to time *t*, and the allele frequency change that takes place between time points *t* and $t+\tau$. We could therefore refer to $-II$ as a correlation between past and present allele frequency change. Extending the argument in Hössjer et al. (2016), where the case $\varvec{w}=\varvec{v}$ was treated, one finds that

23 $$\begin{aligned} II = II_{T+t}(\tau ) = \frac{2\sum _{x,y} ((\varvec{v}\varvec{B}^\tau )_x-w_x)w_y h_{txy}}{\sum _{x,y} w_xw_y h_{txy}}. \end{aligned}$$

It follows from (23) that $II=0$ when all subpopulations are isolated ($\varvec{v}\varvec{B}=\varvec{v}$) and the subpopulation weights are the same at time points *t* and $t+\tau$ ($\varvec{w}=\varvec{v}$). We will therefore often refer to *II* as a migration or gene flow term, since it is impacted by migration in an essential way.

Equation (20) implies that the standardized amount of allele frequency change is non-negative, i.e. $F=I+II\ge 0$. It turns out that the gene flow term *II* is typically non-negative as well, since migration tends to induce a negative correlation between past and present allele frequency change when subpopulations with large allele frequencies receive inflow from other subpopulations with lower frequencies of the same allele. The consequence of such a negative correlation, or positive *II*, is to inflate the expected squared allele frequency change *F*. Since past and present allele frequency changes of a homogeneous population ($s=1$) are uncorrelated, such a population must have $II=0$ and $N_{eV}=N_{eGD}$. The same is true when subpopulations are weighted according to their long term reproductive ability ($\varvec{w}=\varvec{v}=\varvec{\gamma }$), since positive and negative allele frequency changes in different subpopulations will then cancel out in such a way that allele frequency change before time *t* is uncorrelated to the one that takes place over the interval $[t,t+\tau ]$. On the other hand, *II* is typically positive when one subpopulation *x* receives full weight ($\varvec{w}=\varvec{v}=\varvec{e}_x$), and this will lower $N_{eVRx}$ below $N_{eGD}$. The magnitude of *II* for local subpopulation weights depends on the amount of subpopulation differentiation at time *t* (as quantified by $F_{ST,t}$) and the amount of gene flow between the subpopulations. It follows from (14) that this amount of subpopulation differentiation is reflected in terms of how much larger $h_{txy}$ for pairs of different subpopulations $x\ne y$ are compared to all $h_{txx}$. In the extreme case when all elements of $\varvec{h}_t$ are the same it follows that $F_{ST,t}=II=0$ and consequently $N_{eV}=N_{eGD}$. This happens for instance when the first time point *t* of $[t,t+\tau ]$ is the founder generation ($t=-T$). On the other hand, when $II>0$ it may happen that $F=1 \Leftrightarrow II=1-I$ or $F>1 \Leftrightarrow II > 1-I$, which we formally write as $N_{eV}=0$ and $N_{eV}=-\infty$ respectively.

#### Intermediate Approach

The forward definition (20) of the variance effective size relies on a standardized measure $F=I+II$ of expected squared allele frequency change, which sometimes exceeds 1. This is due to the fact that the denominator of *F* in (20) is inflated when the allele frequency at the first time point *t* of the interval $[t,t+\tau ]$ is close to 0 or 1. For this reason, when $N_{eV}$ is estimated from data by the temporal method, allele frequency change is usually standardized in such a way that allele frequencies at both time points *t* and $t+\tau$ are used. In particular, the approach of Pollack (1983) and Jorde and Ryman (2007) corresponds to a definition

24 $$\begin{aligned} F^{\textrm{int}} = \frac{E[(p_{t+\tau }-p_t)^2]}{E[(p_t+p_{t+\tau })/2\cdot (1-(p_t+p_{t+\tau })/2)]} \end{aligned}$$

of standardized expected squared allele frequency change, whose denominator involves allele frequencies $p_t$ and $p_{t+\tau }$ at both time points *t* and $t+\tau$. We refer to (24) as the intermediate version of the standardized expected squared allele frequency change, since the allele frequency change of the numerator is forward *or* backward in time, from the perspective of time point *t* and $t+\tau$ respectively. Let $N_{eV}^{\textrm{int}}$ refer to the corresponding intermediate version of variance effective size that makes use of $F^{\textrm{int}}$ rather than *F*. It is not possible to define $N_{eV}^{\textrm{int}}$ by simply replacing *F* with $F^{\textrm{int}}$ in (20), since for a Wright–Fisher population, such a procedure would not retain the population size. Instead, following Jorde and Ryman (2007) we put

25 $$\begin{aligned} Q(\tau ) = 1 - \left( 1-\frac{1}{2N_{eV}^{\textrm{int}}}\right) ^\tau = \frac{F^{\textrm{int}}}{1+\frac{1}{4}F^{\textrm{int}}}. \end{aligned}$$

It can be seen that the intermediate approach is somewhat more stable than the forward approach. Indeed, the right hand side of (25) is less than 1 whenever $F^{\textrm{int}}<4/3$, so that $N_{eV}^{\textrm{int}}$ exists whenever $0<F^{\textrm{int}}<4/3$.

In order to analyze $N_{eV}^{\textrm{int}}$ more closely, we need an expression for the genetic drift term $F^{\textrm{int}}$ in (24). To this end, we have to replace the denominator $E[p_t(1-p_t)]$ of *F* in (20) by $E[(p_t+p_{t+\tau })/2\cdot (1-(p_t+p_{t+\tau })/2)]$. The ratio of these two denominators is

26 $$\begin{aligned} \frac{{E\left[ {\frac{{p_{t} + p_{{t + \tau }} }}{2}\left( {1 - \frac{{p_{t} + p_{{t + \tau }} }}{2}} \right)} \right]}}{{E[p_{t} (1 - p_{t} )]}} & = \frac{{p(1 - p)}}{{E[p_{t} (1 - p_{t} )]}} - \frac{{E\left( {\frac{{p_{t} + p_{{t + \tau }} }}{2} - p} \right)^{2} }}{{E[p_{t} (1 - p_{t} )]}} \\ & = \frac{{p(1 - p)}}{{E[p_{t} (1 - p_{t} )]}} - \frac{3}{4}\frac{{E[(p_{t} - p)^{2} ]}}{{E[p_{t} (1 - p_{t} )]}}\quad \\ &\quad - \frac{1}{4}\frac{{E[(p_{{t + \tau }} - p)^{2} ]}}{{E[p_{t} (1 - p_{t} )]}} - \frac{1}{2}\frac{{{\text{Cov}}(p_{{t + \tau }} - p_{t} ,p_{t} - p)}}{{E[p_{t} (1 - p_{t} )]}} \\ & = 1 + \frac{1}{4}\frac{{E[(p_{t} - p)^{2} ]}}{{E[p_{t} (1 - p_{t} )]}} - \frac{1}{4}\frac{{E[(p_{{t + \tau }} - p)^{2} ]}}{{E[p_{t} (1 - p_{t} )]}} \\ &\quad - \frac{1}{2}\frac{{{\text{Cov}}(p_{{t + \tau }} - p_{t} ,p_{t} - p)}}{{E[p_{t} (1 - p_{t} )]}} \\ & = 1 - \frac{1}{4}I + \frac{1}{4}II. \\ \end{aligned}$$

Inserting (21) and (26) into (24) we find that

27 $$\begin{aligned} F^{\textrm{int}} = \frac{I + II}{1-\frac{1}{4}I + \frac{1}{4}II}. \end{aligned}$$

When the last equation is plugged into (25), an expression

28 $$\begin{aligned} 1 - \left( 1-\frac{1}{2N_{eV}^{\textrm{int}}}\right) ^\tau = \frac{I + II}{1 + \frac{1}{2}II} \end{aligned}$$

is obtained for the intermediate definition of the variance effective size. From this it follows that the threshold for the intermediate version of the variance effective size not to exist ($N_{eV}^{\textrm{int}}=-\infty$) is twice as high ($II > 2(1-I)$) as compared to the forward version of this effective size.

#### Backward Approach

In analogy with (24) and (25), we also introduce a novel *backward* definition of $N_{eV}$. In the first step expected squared allele frequency change

29 $$\begin{aligned} F^{\textrm{back}} = \frac{E[(p_{t+\tau }-p_t)^2]}{E[p_{t+\tau }(1-p_{t+\tau })]} \end{aligned}$$

is normalized using allele frequencies from the right end point $t+\tau$ of the time interval along which genetic change is monitored. From the horizon of an observer at this time point (29) describes what happened in the past, since the expected squared allele frequency change of the numerator is applied to a time period of the past. Let $N_{eV}^{\textrm{back}}$ denote the variance effective size that makes use of $F^{\textrm{back}}$ rather than *F*. In order for $N_{eV}^{\textrm{back}}$ to retain the size of a Wright–Fisher population, we need to define it as

30 $$\begin{aligned} Q(\tau ) = 1 - \left( 1-\frac{1}{2N_{eV}^{\textrm{back}}}\right) ^\tau = \frac{F^{\textrm{back}}}{1+F^{\textrm{back}}}. \end{aligned}$$

It follows that $N_{eV}^{\textrm{back}}$ exists for all scenarios such that the standardized expected squared allele frequency change between generations *t* and $t+\tau$ satisfies $0<F^{\textrm{back}}< \infty$, since this implies $0<Q(\tau )<1$. For this reason the backward approach (30) gives a more stable definition of variance effective size than the forward and intermediate definitions in (20) and (25).

In order to study $N_{eV}^{\textrm{back}}$ more closely, we start by deriving an expression for $F^{\textrm{back}}$ in (29). To this end, we have to replace the denominator $E[p_t(1-p_t)]$ of *F* in (20) by $E[p_{t+\tau }(1-p_{t+\tau })]$. By similar calculations as in (26), we find that the ratio of these two denominators is

31 $$\begin{aligned} \frac{E[p_{t+\tau }(1-p_{t+\tau })]}{E[p_t(1-p_t)]} = 1 - I. \end{aligned}$$

In a two-step procedure, we first insert (21) and (31) into (29) and find that

32 $$\begin{aligned} F^{\textrm{back}} = \frac{I + II}{1-I}. \end{aligned}$$

When the last equation is plugged into (30), a formula

33 $$\begin{aligned} 1 - \left( 1-\frac{1}{2N_{eV}^{\textrm{back}}}\right) ^\tau = \frac{I + II}{1 + II} \end{aligned}$$

for the backward definition of the variance effective size is derived. It follows that $N_{eV}^{\textrm{back}}$ exists under the very mild requirements $I+II>0$ and $I<1$, since this implies $0<Q(\tau )< 1$.

### Eigenvalue Effective Size

The eigenvalue effective size $N_{eE}$ corresponds to the long term rate at which genetic variability is lost. The formal definition of the eigenvalue effective size is

34 $$\begin{aligned} Q = \lambda = 1 - \frac{1}{2N_{eE}}, \end{aligned}$$

where $\lambda =\lambda _3(\varvec{P})$ is the largest non-unit eigenvalue and the third largest eigenvalue overall of the transition matrix $\varvec{P}$ of $\{\varvec{p}_t=(p_{t1},\ldots ,p_{ts});\, t=-T,-T+1,\ldots \}$, the vector-valued Markov chain of allele frequencies in all subpopulations. This Markov chain is defined on a huge state space of size $\prod _{x=1}^s (2N_{cx}+1)$. Tufto et al. (1996) and Tufto and Hindar (2003) used a slightly different definition of $\lambda$ in (34), as the largest eigenvalue

35 $$\begin{aligned} \lambda = \lambda _{\textrm{max}}(\varvec{A}) \end{aligned}$$

of the much smaller matrix $\varvec{A}$ that appears in the linear recursion for one minus standardized covariances as well as for gene diversities (cf. (3) and (7)). Indeed, by the Perron–Frobenius Theorem $\varvec{A}$ has a unique, real-valued, and positive eigenvalue of multiplicity 1, which is strictly larger than the modulus of all other eigenvalues of $\varvec{A}$. It follows from work of Whitlock and Barton (1997) and Hössjer (2015) that $\lambda _3(\varvec{P})=\lambda _{\textrm{max}}(\varvec{A})$.

### Relations Between Effective Sizes

It is clear from the definition (18) of the gene diversity effective size and the three versions (21), (28), and (33) of the variance effective size that whenever the gene flow term *II* is non-negative ($II\ge 0$) the values of $Q(\tau )$ for these four effective sizes satisfy

$$\begin{aligned} I \le \frac{I+II}{1+II} \le \frac{I+II}{I+\frac{1}{2}II} \le I + II, \end{aligned}$$

making use of the fact that $I\le 1$ because of (18), and that $I+II\ge 0$ must hold as a consequence of (21). But since $N_{eQ}$ is a strictly decreasing function of $Q(\tau )$ in (16), it follows that

36 $$\begin{aligned} N_{eV} \le N_{eV}^{\textrm{int}} \le N_{eV}^{\textrm{back}} \le N_{eGD}. \end{aligned}$$

These inequalities involve the possibility that some effective sizes have values $-\infty$, 0, or $\infty$, whenever $Q(\tau )>1$, $Q(\tau )=1$ and $Q(\tau )\le 0$, as discussed above. There is no general relation between $N_{eE}$ and the four effective sizes in (36). We will find however that under migration–drift equilibrium $N_{eE}$ equals $N_{eGD}$ as well as $N_{eV}$ with reproductive subpopulation weights.

## Migration–Drift Equilibrium

Migration–drift equilibrium occurs when many generations have elapsed between the founder generation and the first generation *t* of the interval over which genetic change is assessed, so that a balance between genetic drift within and migration between subpopulation is obtained. Mathematically, this corresponds to keeping *t* fixed while $T\rightarrow \infty$. Recall from (35) that $\varvec{A}$ has a unique, real-valued, and largest eigenvalue $\lambda$. Let $\varvec{r}=(r_{xy})$ be the corresponding right eigenvector of $\varvec{A}$ with eigenvalue $\lambda$, whose elements, by the Perron–Frobenius Theorem, are real-valued and positive. In view of (5), it follows that $\varvec{h}_{-T}=\varvec{1}_{s^2} = C\varvec{r}+ \varvec{r}^\prime$ for some constant $C> 0$, where $\varvec{r}^\prime$ is a linear combination of the other right eigenvectors of $\varvec{A}$. Consequently, it follows from the linear recursion (3) that

37 $$\begin{aligned} \varvec{h}_t \approx C\lambda ^{t+T}\varvec{r} \end{aligned}$$

is an increasingly accurate approximation as *T* gets large. For this reason the migration–drift properties of the metapopulation will only involve $\lambda$ and $\varvec{r}$.

### Example 1

(Symmetric migration and equally large subpopulations) In order to find more explicit expressions for $\varvec{r}$, we will consider a class of structured populations that includes the island and stepping stone models as special cases. These populations have subpopulations with equally large local census sizes ($N_{cx}=N_c$) and equally large local effective sizes under isolation ($N_{ex}=N_e$). The backward migration rates $B_{xy}$ may depend on the pair *x*, *y* of subpopulations, but it is assumed that they are the same in both directions between any such pair. Consequently, the backward migration matrix $\varvec{B}$ is symmetric ($B_{xy}=B_{yx}$ for all $x\ne y$). Since we also assume that $\varvec{B}$ is irreducible, this implies that an asymptotic distribution $\varvec{\gamma }= \varvec{1}_s^T/s$ exists for the Markov chain with transition matrix $\varvec{B}$, where $\varvec{1}_s=(1,\ldots ,1)^T$ is a column vector of *s* ones. Moreover, $\varvec{B}$ has real-valued eigenvalues $\eta _i$, with

$$\begin{aligned} 1 = \eta _1> \eta _2 \ge \ldots \ge \eta _s > -1. \end{aligned}$$

Let $\varvec{l}_1=\sqrt{s}\varvec{\gamma }=\varvec{1}_s^T/\sqrt{s}, \varvec{l}_2, \ldots ,\varvec{l}_s$ be the corresponding orthonormal system of left eigenvectors $\varvec{l}_i$ of $\varvec{B}$, expressed as $\varvec{l}_i=(l_{ix}; \, x=1,\ldots ,s)$. It is shown in Appendix B.1 that the column vectors $\varvec{l}_{ij}^T = (l_{ix}l_{jy}; \, 1\le x,y \le s)^T$ of length $s^2$ form a convenient orthonormal system of basis functions to use in order to analyze the right eigenvector $\varvec{r}$ of $\varvec{A}$, for a system with symmetric migration.

The island model is an instance of symmetric migration, with

38 $$\begin{aligned} \varvec{B}= (1-m)\varvec{I}_s + m\varvec{1}_s \varvec{1}_s^T, \end{aligned}$$

and $\varvec{I}_s$ the identity matrix of order *s*. The non-unit eigenvalues of this migration matrix are

39 $$\begin{aligned} \eta _2=\ldots =\eta _s=1-m. \end{aligned}$$

The circular stepping stone model is a second example of symmetric migration, where any subpopulation *x* receives a fraction *m*/2 of genes from each of its two neighboring subpopulations $x-1$ and $x+1$ modulo *s*. This corresponds to a backward migration matrix

40 $$\begin{aligned} \varvec{B}= \left( \begin{array}{ccccc} 1-m &{} m/2 &{} 0 &{} \ldots &{} m/2 \\ m/2 &{} 1-m &{} m/2 &{} \ldots &{} 0\\ \vdots &{} &{} &{} \ddots &{} \vdots \\ m/2 &{} 0 &{} \ldots &{} m/2 &{} 1-m \end{array}\right) . \end{aligned}$$

The matrix in (40) is a circular matrix, and Fourier analysis of such matrices has frequently been used in population genetics (Malécot 1951; Maruyama 1970a; Rousset 2004; Hössjer 2014). For instance, it is shown in Hössjer (2014) that

41 $$\begin{aligned} \eta _i = 1-m + m\cos \left( \frac{2\pi [(i+1)/2]}{s}\right) , \quad i=1,\ldots ,s, \end{aligned}$$

with $[(i+1)/2]$ the integer part of $(i+1)/2$. Expressions for $\eta _i$ for the two-dimensional (torus) stepping stone model can be found in Hössjer (2014). $\square$

### Subpopulation Differentiation

In order to find an expression for the fixation index $F_{ST,t}$ under migration–drift equilibrium, we insert (37) into (14) and let $T\rightarrow \infty$. This yields

42 $$\begin{aligned} F_{ST,t} {\mathop {\rightarrow }\limits ^{T\rightarrow \infty }} F_{ST}^{\textrm{eq}} = \frac{\sum _{x,y} w_x w_y r_{xy} - \sum _x w_x r_{xx}}{\sum _{x,y} w_x w_y r_{xy}}, \end{aligned}$$

where superscript eq is an acronym for equilibrium. It is shown in Appendix B.2 that for reproductive weights $\varvec{w}=\varvec{\gamma }$ and the symmetric model of Example 1, the approximation

43 $$\begin{aligned} F_{ST}^{\textrm{eq}} \approx \frac{s-1}{2s} \left( \frac{1}{N_c} + \frac{1}{N_e}\cdot \frac{1}{s-1}\sum _{i=2}^s\frac{\eta _i^2}{1-\eta _i^2}\right) \end{aligned}$$

is accurate for large local population sizes when subpopulations are connected by strong migration. For the island model (43) we insert (39) into (43) and obtain

44 $$\begin{aligned} F_{ST}^{\textrm{eq}} \approx \frac{s-1}{2s\tilde{N} [1-(1-m)^2]}, \end{aligned}$$

where

45 $$\begin{aligned} \frac{1}{\tilde{N}} = \frac{1-(1-m)^2}{N_c} + \frac{(1-m)^2}{N_e} \end{aligned}$$

is a harmonic average of the local census and effective sizes. Formula (44) is accurate when *m* is not too small. For improved island model approximations of $F_{ST}^{\textrm{eq}}$, see Hössjer et al. (2013).

### Genetic Drift and Migration

Next we will analyze how the genetic drift term $I=I_{T+t}(\tau )$ and the gene flow term $II=II_{T+t}(\tau )$ behave as $T\rightarrow \infty$. From (3), (22), and (37) we deduce that

46 $$\begin{aligned} I {\mathop {\rightarrow }\limits ^{T\rightarrow \infty }} I_\infty (\tau ) = 1 - \left( 1-\frac{1}{2N_{eE}}\right) ^\tau \end{aligned}$$

and

47 $$\begin{aligned} II {\mathop {\rightarrow }\limits ^{T\rightarrow \infty }} II_\infty (\tau ) = \frac{2\sum _{x,y} ((\varvec{v}\varvec{B}^\tau )_x-w_x)w_y r_{xy}}{\sum _{x,y} w_xw_y r_{xy}} \end{aligned}$$

when *t* and $t+\tau$ are kept fixed while $T\rightarrow \infty$.

It is shown in Appendix B.3 that the equilibrium gene flow term (47) simplifies to

48 $$\begin{aligned} II_\infty (\tau ) \approx \sum _{i=2}^s \frac{\kappa _i(\kappa _i-\eta _i^\tau \rho _i)}{1-\eta _i^2} \left( \frac{1-\eta _i^2}{N_c} + \frac{\eta _i^2}{N_e}\right) \end{aligned}$$

for symmetric migration (cf. Example 1), with $\kappa _i = \varvec{w}\varvec{l}_i^T$ and $\rho _i=\varvec{v}\varvec{l}_i^T$ the coefficients of $\varvec{l}_i$ for $\varvec{w}$ and $\varvec{v}$, when these two weight vectors are expanded as a linear combination of the left eigenvectors $\varvec{l}_i$ of $\varvec{B}$. Formula (48) is accurate when the subpopulations are connected by strong migration. For the island model (38) and (39) we have that $\sum _{i=2}^s \kappa _i^2 = \sum _{x=1}^s w_x^2 - \kappa _1^2 = |\varvec{w}|^2 - 1/s$ and $\sum _{i=2}^s \kappa _i\rho _i = \sum _x w_xv_x - \kappa _1\rho _1 = \varvec{w}\varvec{v}^T - 1/s$. Then (48) simplifies to

49 $$\begin{aligned} II_\infty (\tau ) \approx \frac{|\varvec{w}|^2-1/s - (1-m)^\tau (\varvec{w}\varvec{v}^T - 1/s)}{\tilde{N}[1-(1-m)^2]}, \end{aligned}$$

with $\tilde{N}$ as in (45). This formula is accurate as long as *m* is not too small. In particular, if $1\le k \le s$ subpopulations receive equal weight 1/*k* at time points *t* and $t+\tau$, and $\max (2k-s,0) \le l \le k$ of these overlap, it follows that $|\varvec{w}|^2 = 1/k$ and $\varvec{w}\varvec{v}^T = l/k^2$. Insertion into (49) gives

50 $$\begin{aligned} II_\infty (\tau ) \approx \frac{1/k-1/s - (1-m)^\tau (l/k^2 - 1/s)}{\tilde{N}[1-(1-m)^2]}. \end{aligned}$$

Notice in particular that the right hand side of (50) vanishes when $k=l=s$. This corresponds to using equal weights $w_x=v_x=1/s$ of all subpopulations at both time points *t* and $t+\tau$, which are the reproductive weights for the island model.

In Sects. 6.3 and 6.4 we will use (46)–(50) in order to derive explicit expressions for the gene diversity and variance effective sizes under migration–drift equilibrium.

### Gene Diversity Effective Size

It follows from (18) and (46) that the gene diversity effective size equals the eigenvalue effective size under migration–drift equilibrium, since

51 $$\begin{aligned} N_{eGD} {\mathop {\rightarrow }\limits ^{T\rightarrow \infty }} N_{eGD}^{\textrm{eq}} = N_{eE}. \end{aligned}$$

Notice in particular that since the equilibrium limit $I_\infty (\tau )$ of the drift term in (46) does not involve the subpopulation weighting scheme $\varvec{w}$, (51) holds regardless which $\varvec{w}$ we use to define $N_{eGD}$.

### Variance Effective Size

#### Forward Approach

The two equations (46) and (47) have interesting implications for the asymptotic limit of the forward version of the variance effective size $N_{eV}$ at migration–drift equilibrium. It follows from (20), (21), (46), and (47) that

52 $$\begin{aligned} N_{eV} {\mathop {\rightarrow }\limits ^{T\rightarrow \infty }} N_{eV}^{\textrm{eq}} = \frac{1}{2\{1-[(1-1/(2N_{eE}))^\tau - II_\infty (\tau )]^{1/\tau }\}} \end{aligned}$$

for all $\tau$ such that $0<I_\infty (\tau )+II_\infty (\tau )<1$, or equivalently that

53 $$\begin{aligned} \left( 1-\frac{1}{2N_{eE}}\right) ^\tau > II_\infty (\tau ) \end{aligned}$$

holds. We may apply (52) to any kind of weighting scheme. Since $N_{eVMeta}$ is based on reproductive weights $\varvec{w}=\varvec{v}=\varvec{\gamma }$, and $\varvec{\gamma }=\varvec{\gamma }\varvec{B}$, it follows from (23) that $II_{T+t}(\tau )=0$ for any $T\ge 0$, and hence $II_\infty (\tau )=0$. Insertion into (52) gives

54 $$\begin{aligned} N_{eVMeta} {\mathop {\rightarrow }\limits ^{T\rightarrow \infty }} N_{eVMeta}^{\textrm{eq}} = N_{eE}. \end{aligned}$$

For local subpopulation weights we insert $\varvec{v}=\varvec{w}=\varvec{e}_x$ into the definition of $II_\infty (\tau )$ in (47). In conjunction with (52) this gives the equilibrium value $N_{eVRx}^{\textrm{eq}}$ of the realized variance effective size of subpopulation *x*, for all $\tau$ such that (53) holds.

It is proved in Appendix B.4 that the variance effective size at migration–drift equilibrium satisfies

55 $$\begin{aligned} N_{eV\varvec{w}\varvec{v}}^{\textrm{eq}} \le N_{eV\varvec{w}}^{\textrm{eq}} \end{aligned}$$

for the island model, and subpopulations weights $\varvec{w}$ and $\varvec{v}$ at time points *t* and $t+\tau$ such that $\varvec{w}\varvec{v}^T\le |\varvec{w}|^2$, with equality in (55) if and only if $\varvec{w}\varvec{v}^T=|\varvec{w}|^2$. The intuition behind (55) is that $II_\infty (\tau )$ is elevated when different subpopulation weights are used in generations *t* and $t+\tau$, since the negative correlation *II* between allele frequency change of the past and present then increases, so that the variance effective size gets smaller. We also verify in Appendix B.4 that

56 $$\begin{aligned} N_{eV\varvec{w}}^{\textrm{eq}} \le N_{eV\varvec{\gamma }}^{\textrm{eq}} = N_{eVMeta}^{\textrm{eq}} = N_{eE} \end{aligned}$$

for the symmetric migration models of Example 1, with equality if and only if reproductive weights ($\varvec{w}=\varvec{v}=\varvec{\gamma }$) are used at time points *t* and $t+\tau$. The intuition behind (56) is that the gene flow term $II_\infty (\tau )$ is positive as soon as non-reproductive weights $\varvec{w}=\varvec{v}\ne \varvec{\gamma }$ are used, so that $N_{eV\varvec{w}}$ gets smaller. We also conjecture that results similar to (55) and (56) hold more generally than for island and symmetric migration models respectively.

It is instructive to illustrate (55) and (56) for an island model where $1\le k \le s$ subpopulations are assigned equal weight 1/*k* at both time points *t* and $t+\tau$, and that *l* of these subpopulations overlap. Insertion of the equilibrium migration term $II_\infty (\tau )$ in (50) into (52) yields

57 $$\begin{aligned} N_{eV}^{\textrm{eq}} \approx \frac{1}{2\left\{ 1-\left[ (1-1/(2N_{eE}))^\tau - \frac{1/k-1/s - (1-m)^\tau (l/k^2 - 1/s)}{\tilde{N}[1-(1-m)^2]}\right] ^{1/\tau }\right\} }. \end{aligned}$$

This formula shows very explicitly how much $N_{eV}^{\textrm{eq}}$ differs from $N_{eE}$, as a function of *k* and *l*. For fixed *k*, $N_{eV}^{\textrm{eq}}$ is maximized in (57) when the same subpopulation weights are used at both time points ($l=k$), in agreement with (55). When $k=l$, we notice that $N_{eV}^{\textrm{eq}}$ attains its maximum $N_{eE}$ when reproductive weights are used at both time points, which corresponds to $k=s$ and $\varvec{w}=\varvec{\gamma }=\varvec{1}_s^T/s$, in agreement with (56).

#### Intermediate Approach

For the intermediate approach, we have, analogously to (52), that the variance effective size at equilibrium is

58 $$\begin{aligned} N_{eV}^{\textrm{int}} {\mathop {\rightarrow }\limits ^{T\rightarrow \infty }} N_{eV}^{\textrm{int,eq}} = \frac{1}{2\left\{ 1-\left[ \frac{(1-1/(2N_{eE}))^\tau - \frac{1}{2}II_\infty (\tau )}{1+\frac{1}{2}II_\infty (\tau )}\right] ^{1/\tau }\right\} }, \end{aligned}$$

for all $\tau$ such that

59 $$\begin{aligned} \left( 1-\frac{1}{2N_{eE}}\right) ^\tau > \frac{1}{2}II_\infty (\tau ), \end{aligned}$$

which is a less stringent condition than (53) for the variance effective size to exist. Since $II_\infty (\tau )=0$ for reproductive weights, it follows that $N_{eVMeta}^{\textrm{int,eq}}$ converges to $N_{eE}$ as migration–drift equilibrium is approached, as in (54). The local realized variance effective size $N_{eVRx}^{\textrm{int,eq}}$ at equilibrium is obtained by inserting $\varvec{w}=\varvec{v}=\varvec{e}_x$ into the definition of $II_\infty (\tau )$ in (58). Formulas (55) and (56) hold for the intermediate version of the variance effective size as well, and explicit expressions of $N_{eV}^{\textrm{int,eq}}$ for the island model are obtained by inserting (50) into (58).

#### Backward Approach

For the backward approach, we find that the variance effective size at equilibrium exists for time intervals of any length $\tau$. This equilibrium value

60 $$\begin{aligned} N_{eV}^{\textrm{back}} {\mathop {\rightarrow }\limits ^{T\rightarrow \infty }} N_{eV}^{\textrm{back,eq}} = \frac{1}{2\left\{ 1-\frac{1-1/(2N_{eE})}{[1+II_\infty (\tau )]^{1/\tau }}\right\} } \end{aligned}$$

is derived in the same way as (52) and (58). For local subpopulation weights we insert $\varvec{w}=\varvec{v}=\varvec{e}_x$ into the definition of $II_{\infty }(\tau )$ in Eq. (60) in order to obtain $N_{eV,Rx}^{\textrm{back,eq}}$. Formulas (55) and (56) hold for the backward version of the variance effective size as well, and explicit expressions of $N_{eV}^{\textrm{back,eq}}$ for the island model are obtained by inserting (50) into (60).

## The Length of the Time Interval

In this section we analyze how the length $\tau$ of the time interval impacts the gene diversity and variance effective sizes. We will focus on the two extreme scenarios of consecutive generations ($\tau =1$) and long time intervals ($\tau \rightarrow \infty$).

### Consecutive Generations

For ease of notation, we will sometimes write $I_{T+t}(\tau )=I(\tau )$ and $II_{T+t}(\tau )=II(\tau )$ for the genetic drift and gene flow terms that appear in the definitions of the gene diversity and variance effective sizes. When these effective sizes reflect changes between two consecutive generations ($\tau = 1$), formulas (18), (21), (28), and (33) simplify to

61 $$\begin{aligned} N_{eGD}= &{} \frac{1}{2I(1)} {\mathop {=}\limits ^{\textrm{eq}}}& N_{eE}, \end{aligned}$$

62 $$\begin{aligned} N_{eV}= & {} \frac{1}{2[I(1)+II(1)]} {\mathop {=}\limits ^{\textrm{eq}}} \frac{1}{\frac{1}{N_{eE}} + 2II_\infty (1)}, \end{aligned}$$

63 $$\begin{aligned} N_{eV}^{\textrm{int}}= & {} \frac{1 + \frac{1}{2} II(1)}{2[I(1)+II(1)]} {\mathop {=}\limits ^{\textrm{eq}}} \frac{1 + \frac{1}{2} II_\infty (1)}{\frac{1}{N_{eE}} + 2II_\infty (1)}, \end{aligned}$$

and

64 $$\begin{aligned} N_{eV}^{\textrm{back}} = \frac{1 + II(1)}{2[I(1)+II(1)]} {\mathop {=}\limits ^{\textrm{eq}}} \frac{1 + II_\infty (1)}{\frac{1}{N_{eE}} + 2II_\infty (1)} \end{aligned}$$

respectively, where the right hand sides of (61)–(64) refer to migration–drift equilibrium, with $I_\infty (1)$ and $II_\infty (1)$ the drift and gene flow terms in (46) and (47) at equilibrium, for two consecutive generations. Typically the gene flow term is small ($II(1)\ll 1$) unless there is much migration between the subpopulations and a large amount of subpopulation differentiation at time *t*. Consequently, for most scenarios of practical interest the three versions of variance effective size are practically the same,

65 $$\begin{aligned} \frac{1}{2[I(1)+II(1)]} = N_{eV} \approx N_{eV}^{\textrm{int}} \approx N_{eV}^{\textrm{back}}, \end{aligned}$$

when the expected squared allele frequency change between two consecutive generations is analyzed. This is illustrated in Tables 2 and 3 for the realized local variance effective size of an island model with $s=10$ subpopulations at migration–drift equilibrium, corresponding to the right hand side of (62)–(64). Whereas the same subpopulation weights are used at both time points in Table 2 ($\varvec{w}=\varvec{v}=\varvec{e}_x$ or $k=l=1$), this is not the case in Table 3 ($\varvec{w}=\varvec{e}_x$, $\varvec{v}=\varvec{e}_y$, $x\ne y$ or $k=1$, $l=0$). Note in particular that all three versions of the realized local variance effective size depend strongly on the local census size. This phenomenon is discussed in Ryman et al. (2023), and in the present framework in can be explained as follows: The term $II_{\infty }(1)$ is approximated by (49) and (50) for the island model and it depends on the amount of migration into *x* or *y* from the other subpopulations as well as the amount of subpopulation differentiation $F_{ST}^{\textrm{eq}}$ in (44). The larger the migration rate and the local census size are, the smaller is the amount of subpopulation differentiation, and the smaller is the gene flow term $II_\infty (1)$ at equilibrium, so that the variance effective size approaches the eigenvalue effective size.

**Table 2:** Values of the realized local variance effective size $N_{eVRx}$ at migration–drift equilibrium for a time interval of length $\tau =1$, so that the same subpopulation *x* receives full weight at the two end points of the interval

	$N_{cx}=50$			$N_{cx}=500$		
	For	Int	Back	For	Int	Back
0.1	47.2960	47.5232	47.7504	56.4109	56.6337	56.8566
0.2	45.0331	45.2609	45.4888	64.3622	64.5805	64.7988
0.3	42.7467	42.9755	43.2043	74.2336	74.4468	74.6600
0.4	40.4368	40.6667	40.8966	86.5645	86.7714	86.9783
0.5	38.1032	38.3342	38.5652	101.9387	102.1379	102.3371
0.6	35.7455	35.9777	36.2098	120.7432	120.9329	121.1227
0.7	33.3633	33.5967	33.8300	142.4849	142.6637	142.8426
0.8	30.9564	31.1909	31.4254	164.3497	164.5176	164.6854
0.9	28.5242	28.7599	28.9956	179.5012	179.6615	179.8217
1.0	26.0664	26.3033	26.5403	178.4566	178.6174	178.7781

An island model with $s=10$ subpopulations is used, with local effective size $N_{ex}=50$ under isolation, local census size $N_{cx}$ and migration parameter *m* (where $B_{xy}=m/s$ when $x\ne y$ and $B_{xx}=1-(s-1)m/s$). The three methods of computing $N_{eVRx}$ refer to the forward approach (= For, the right hand side of (62)), the intermediate approach (= Int, the right hand side of (63)), and the backward approach (= Back, the right hand side of (64)). A more explicit approximation of $N_{eVRx}$, for the forward approach, appears in (66)

**Table 3:** Values of the realized local variance effective size $N_{eVRxy}$ at migration–drift equilibrium, for a time interval of length $\tau =1$, so that different subpopulations *x* and *y* receive full weight at the two end points of the interval

*m*	$N_{cx}=50$			$N_{cx}=500$		
	For	Int	Back	For	Int	Back
0.1	4.6878	4.9355	5.1833	5.6902	5.9375	6.1847
0.2	8.9166	9.1622	9.4078	13.1853	13.4288	13.6723
0.3	12.6738	12.9175	13.1612	23.1186	23.3572	23.5957
0.4	15.9668	16.2089	16.4509	36.3781	36.6100	36.8419
0.5	18.8007	19.0413	19.2820	54.0715	54.2945	54.5176
0.6	21.1778	21.4172	21.6567	77.2921	77.5035	77.7149
0.7	23.0977	23.3362	23.5746	106.2835	106.4804	106.6774
0.8	24.5571	24.7948	25.0326	138.5156	138.6964	138.8772
0.9	25.5498	25.7870	26.0242	166.3374	166.5042	166.6710
1.0	26.0664	26.3033	26.5403	178.4566	178.6174	178.7781

An island model with $s=10$ subpopulations is used, with local effective size $N_{ex}=50$ under isolation, local census size $N_{cx}$ and migration parameter *m* (where $B_{xy}=m/s$ when $x\ne y$ and $B_{xx}=1-(s-1)m/s$). The three methods of computing $N_{eVRxy}$ refer to the forward approach (=For, the right hand side of (62)), the intermediate approach (=Int, the right hand side of (63)), and the backward approach (=Back, the right hand side of (64)). A more explicit approximation of $N_{eVRxy}$, for the forward approach, appears in (67)

A more analytical interpretation of the results of Table 2 is obtained by inserting $\tau =1$ and $\varvec{w}=\varvec{v}=\varvec{e}_x$ into (49) and the right hand side of (62). This yields

66 $$\begin{aligned} N_{eVRx}^{\textrm{eq}} \approx \frac{1}{\frac{1}{N_{eE}} + \frac{2(1-1/s)}{(2-m)\tilde{N}}} {\mathop {\approx }\limits ^{(44)}} \frac{1}{\frac{1}{N_{eE}} + 4m F_{ST}^{\textrm{eq}}}. \end{aligned}$$

On the other hand, in order to approximate the results of Table 3, we insert $\tau =1$, $\varvec{w}=\varvec{e}_x$, and $\varvec{v}=\varvec{e}_y$ into the right hand side of (62). This yields

67 $$\begin{aligned} N_{eVRxy}^{\textrm{eq}} \approx \frac{1}{\frac{1}{N_{eE}} + \frac{2(1-m/s)}{(2-m)m\tilde{N}} } {\mathop {\approx }\limits ^{(44)}} \frac{1}{\frac{1}{N_{eE}} + 4F_{ST}^{\textrm{eq}}\frac{s-m}{s-1}}. \end{aligned}$$

Formulas (66) and (67) are also obtained from (57), with $\tau =1$, $k=1$, and $l=1$ or $l=0$ respectively. They are accurate when the migration rate *m* is not too small. In particular, under panmixia it follows from (57) that

68 $$\begin{aligned} N_{eV}^{\textrm{eq}} {\mathop {=}\limits ^{m=1}} \frac{1}{\frac{1}{N_{eE}} + \frac{2(1-1/s)}{N_c}}, \end{aligned}$$

regardless of the loal weight vectors $\varvec{w}=\varvec{e}_x$ and $\varvec{v}=\varvec{e}_y$ of the subpopulations at time points *t* and $t+1$. Note that (68) is the limit of (66) and (67) when $m\rightarrow 1$, and that (68) approaches $N_{eE}$ when $N_c\rightarrow \infty$.

### Long Time Intervals

When the length $\tau$ of the time interval gets large, it may happen that the standardized allele frequency change of the forward and intermediate versions of the variance effective size satisfy $Q(\tau )\ge 1$, so that the corresponding effective size equals 0 or $-\infty$. In this subsection we will provide formulas for the maximal length $\tau _{\textrm{max},Q}$ of the time interval for which each type *Q* of effective size exists under migration–drift equilibrium. Since the gene diversity effective size equals the eigenvalue effective size at equilibrium, for time intervals of any length (cf. (51)), it follows that

$$\begin{aligned} \tau _{\textrm{max},GD} = \infty . \end{aligned}$$

For the forward version of the variance effective size, it follows from (53) that

69 $$\begin{aligned} \tau _{\textrm{max},V} \approx \log [II_\infty ^{-1}] \cdot 2N_{eE}, \end{aligned}$$

where the right hand side is interpreted as plus infinity whenever

70 $$\begin{aligned} II_\infty =\lim _{\tau \rightarrow \infty } II_\infty (\tau ) = \frac{2\sum _{x,y} (\gamma _x-w_x)w_y r_{xy}}{\sum _{x,y} w_xw_y r_{xy}} \end{aligned}$$

is zero or negative. The approximation in (69) is accurate for large $N_{eE}$. It implies that $N_{eV}^{\textrm{eq}}$ exists for time intervals up to a maximal length that is proportional to $N_{eE}$. For the intermediate version of the variance effective size, we similarly deduce

71 $$\begin{aligned} \tau _{\textrm{max},V}^{\textrm{int}} \approx \log \big [2II_\infty ^{-1}\big ] \cdot 2N_{eE} \end{aligned}$$

from formula (59). We recall from (60) that the backward version of the variance effective size exists at equilibrium for time intervals of any length, so that

$$\begin{aligned} \tau _{\textrm{max},V}^{\textrm{back}} = \infty . \end{aligned}$$

In particular, by increasing the length of the time interval in (60) we find that

72 $$\begin{aligned} N_{eV}^{\textrm{back,eq}} {\mathop {\rightarrow }\limits ^{\tau \rightarrow \infty }} N_{eE}, \end{aligned}$$

for all types of subpopulation weights $\varvec{w}$.

**Fig. 1:**
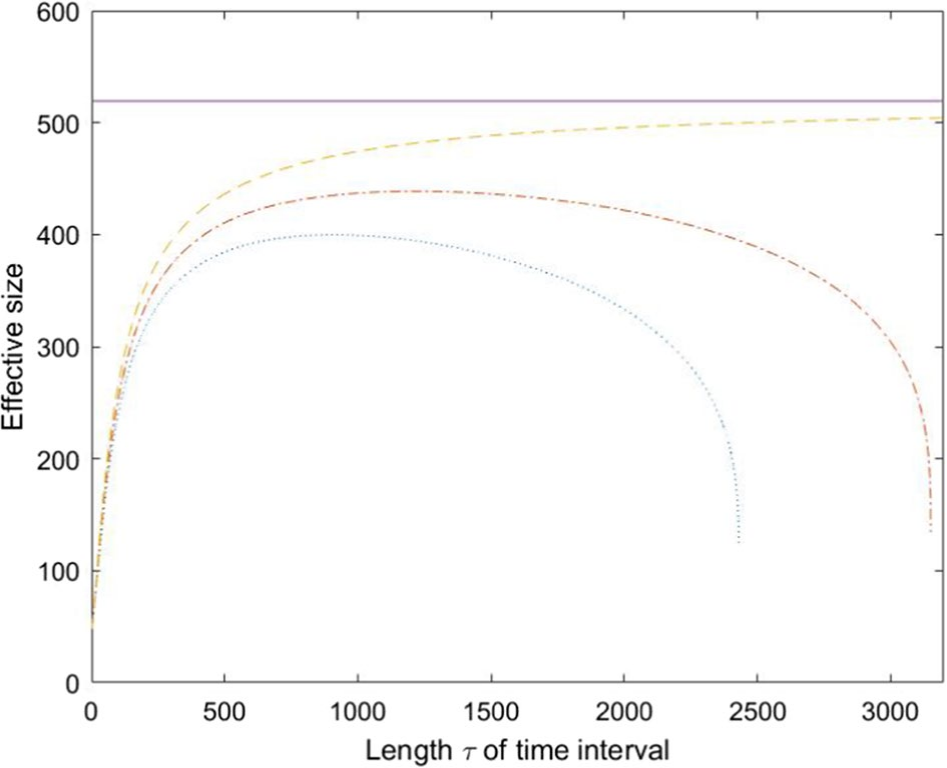
The figure plots effective sizes for an island model with $s=10$, $N_{ex}=N_{cx}=50$ and $m=0.1$, when $t=0$ corresponds to migration–drift equilibrium ($T\rightarrow \infty$). The horizontal solid line corresponds to $N_{eE}=519.28$. The three curves correspond to $N_{eVRx}$ (dotted), $N_{eVRx}^{\textrm{int}}$ (dash-dotted) and $N_{eVRx}^{\textrm{back}}$ (dashed) for intervals $[0,\tau ]$ of increasing length. $N_{eVRx}$ increases with $\tau$ at first, then it starts to drop until $\tau =\tau _{\textrm{max}}=2431$, and for longer intervals $N_{eVRx}$ does not exist. In comparison, formula (69) predicts $\tau _{\textrm{max,V}}=\log (II_\infty ^{-1})\cdot 2N_{eE}= 2432.8$. In a similar fashion $N_{eVRx}^{\textrm{int}}$ increases with $\tau$ at first, then it starts to drop until $\tau =\tau _{\textrm{max}}=3151$, and after this generation $N_{eVRx}^{\textrm{int}}$ does not exist. In comparison, formula (71) predicts $\tau _{\textrm{max},V}^{\textrm{int}}=\log (2II_\infty ^{-1})\cdot 2N_{eE}= 3152.7$. On the other hand, $N_{eVRx}^{\textrm{back}}$ increases monotonically to $N_{eE}$ as $\tau \rightarrow \infty$

Figure 1 illustrates the eigenvalue effective size $N_{eE}$, and the forward, intermediate, and backward versions of the realized local variance effective size over time intervals $[0,\tau ]$ of increasing length when the population is at migration–drift equilibrium. The model is an island model with $s=10$ subpopulations and the migration rate equals $m=0.1$. It can be seen that $N_{eVRx}$ and $N_{eVRx}^{\textrm{int}}$ initially increase as $\tau$ grows, until they reach a maximum, start to decline and eventually do not exist. In contrast, $N_{eVRx}^{\textrm{back}}$ always exists and increases monotonically to $N_{eE}$ as the length $\tau$ of the time interval grows, in agreement with (72). The corresponding variance effective sizes $N_{eVMeta}$, $N_{eVMeta}^{\textrm{int}}$ and $N_{eVMeta}^{\textrm{back}}$ of the metapopulation, based on equal subpopulation weights $w_x=1/s$, equal $N_{eE}$ for all values of $\tau$.

The three realized local variance effective sizes of Fig. 1 are almost the same for time intervals of length up to 200–300 generations, which is at least tenfold the time span typically employed in the context of genetic conservation. However, it is shown in Appendix A that for a small and subdivided population, the three effective sizes may differ substantially for time intervals of length 5–10 generations. More generally, the value of $\tau$ for which the three effective sizes significantly start to differ is proportional to $N_{eE}$. For populations that are either very small locally or experience a severe bottleneck, it may therefore be of interest to use the most stable version $N_{eVRx}^{\textrm{back}}$ of the realized local variance effective size.

## Estimation of Effective Sizes

In this section we will investigate how the length $\tau$ of the time interval impacts the accuracy of an estimator of the gene diversity and variance effective sizes at equilibrium ($N_{eQ}^{\textrm{eq}}$, $Q\in \{GD,V\}$). Our theoretical analysis is complementary to the simulation results of Luikart et al. (1999), where interval lengths with maximal accuracy, for a variance effective size estimator, were derived for a population going through a bottleneck. Here we stick to the model introduced in Sect. 2, with time-invariant population sizes of all subpopulations. We start by introducing

$$\begin{aligned} Q(\tau ) = Q^{\textrm{eq}}(\tau ) = g\left( \frac{1}{2N_{eQ}^{\textrm{eq}}}\right) = 1 - \left( 1-\frac{1}{2N_{eQ}^{\textrm{eq}}}\right) ^\tau , \end{aligned}$$

the value, at migration–drift equilibrium, of the quantity *Q* used to define each effective size. More specifically, in this section $Q(\tau )$ corresponds to the limit, when $T\rightarrow \infty$, of the quantities that appear in (18), (20), (25), and (30) respectively. A necessary requirement for $N_{eQ}^{\textrm{eq}}$ to exist is that $0<Q(\tau )<1$. Recall from Sect. 7.2 that this is not always the case for the forward and intermediate versions of the variance effective size.

In order to estimate $N_{eQ}^{\textrm{eq}}$ from data, let $\hat{Q}(\tau )=Q(\tau )+\varepsilon$ be an estimate of $Q(\tau )$, based on samples of sizes $n_t$ and $n_{t+\tau }$ at time points *t* and $t+\tau$. We will assume that the estimation error $\varepsilon$ of $\hat{Q}(\tau )$ is a random variable with $E(\varepsilon )=0$ and $\text{ Var }(\varepsilon )=\sigma ^2$. Typically $\sigma ^2$ is inversely proportional to the number of biallelic markers used to estimate $Q(\tau )$, with a proportionality constant that is a monotone increasing function of $1/(2n_t)$ and $1/(2n_{t+\tau })$ (Waples 1989). Our objective is to estimate the asymptotic amount of genetic drift

73 $$\begin{aligned} Q = \frac{1}{2N_{eQ}^{\textrm{eq}}} = h(Q(\tau )) \end{aligned}$$

per generation at equilibrium, where

$$\begin{aligned} h(Q(\tau ))=g^{-1}(Q(\tau ))=1-(1-Q(\tau ))^{1/\tau } \end{aligned}$$

is the inverse of *g*. By a first order Taylor expansion of *h*, it can be seen that the error of the estimate $\hat{Q}= h(\hat{Q}(\tau ))$ has an approximate variance

74 $$\begin{aligned} \text{ Var }(\hat{Q}) \approx \sigma ^2 \left( \frac{dh(Q(\tau ))}{dQ(\tau )}\right) ^2 = \sigma ^2 \left\{ \frac{1}{\tau }\left[ 1 - Q(\tau )\right] ^{\frac{1}{\tau }-1}\right\} ^2. \end{aligned}$$

Our objective is to express $\text{ Var }(\hat{Q})$ as a function of $\tau$ for each quantity *Q* and weighting scheme $\varvec{w}$. The variance in (74) will initially decrease with $\tau$, since for short time intervals $Q(\tau )\ll 1$ and consequently

75 $$\begin{aligned} \text{ Var }(\hat{Q}) \approx \frac{\sigma ^2}{\tau ^2}. \end{aligned}$$

When $\tau$ gets larger and $Q(\tau )$ approaches 1, the variance in (74) will reach a minimum and then start to increase. For this reason it is of interest to find approximate expressions for the interval length

76 $$\begin{aligned} \begin{array}{rcl} \tau _{\textrm{opt},Q} &{}=&{} \text{ arg } \min _\tau \text{ Var }(\hat{Q})\\ &{}=&{} \text{ arg }\min _\tau dh(Q(\tau ))/dQ(\tau )\\ &{}=&{} \text{ arg }\min _\tau \frac{1}{\tau }\left[ 1 - Q(\tau )\right] ^{\frac{1}{\tau }-1} \end{array} \end{aligned}$$

that minimizes the estimation variance in (74). As we will find below, $\text{ Var }(\hat{Q})$ is a function of $N_{eE}$ and the equilibrium gene flow term $II_\infty (\tau )$, defined in (47). It turns out that the optimal time interval will have a length $\tau _{\textrm{opt},Q}$ that is proportional to $N_{eE}$. For a system with strong migration between its subpopulations (Nagylaki 1980) $II_\infty (\tau )$ approaches the asymptotic limit (70) so quickly that the length of the transient period is small in comparison to $N_{eE}$. For a population with strong migration we will therefore approximate $\tau _{\textrm{opt},Q}$ by minimizing a simplified version of $dh(Q(\tau ))/dQ(\tau )$ with respect to $\tau$, where $II_\infty (\tau )$ is replaced by the constant $II_\infty$ in (70).

As a complement to (76) we also define

77 $$\begin{aligned} \tau _{C,Q} = \min \{\tau ; \, \sqrt{\frac{\text{ Var }(\hat{Q})}{\sigma ^{2}}} \ge C\} - 1 \end{aligned}$$

as the largest value of $\tau$ for which the standard deviation of the estimate of *Q* has not exceeded $\sigma$ by a factor of at least $C>1$. In particular, $\tau _{\infty ,Q}$ is closely related to $\tau _{\textrm{max},Q}$.

### Gene Diversity Effective Size

For the gene diversity effective size we recall from (18) and (46) that

$$\begin{aligned} Q(\tau ) = I_\infty (\tau ) = 1 - \left( 1 - \frac{1}{2N_{eE}}\right) ^\tau . \end{aligned}$$

Insertion of this equation into (74) yields

78 $$\begin{aligned} \frac{dh(Q(\tau ))}{dQ(\tau )} = \frac{1}{\tau } \left[ 1 - I_\infty (\tau )\right] ^{\frac{1}{\tau }-1} = \frac{1}{\tau } \left( 1-\frac{1}{2N_{eE}}\right) ^{1-\tau }. \end{aligned}$$

Minimizing (78) with respect to $\tau$, we find that the optimal length of the time interval, when estimating the gene diversity effective size, is

79 $$\begin{aligned} \tau _{\textrm{opt},GD} = 2N_{eE}. \end{aligned}$$

### Variance Effective Size

#### Forward Approach

The quantity $Q(\tau )$ of the forward version of the variance effective size is obtained from (20) and (46) and (47). The resulting formula

80 $$\begin{aligned} Q(\tau ) = 1 - \left( 1-\frac{1}{2N_{eE}}\right) ^\tau + II_\infty (\tau ) \end{aligned}$$

leads to

81 $$\begin{aligned} \frac{dh(Q(\tau ))}{dQ(\tau )} = \frac{1}{\tau }\left[ 1 - Q(\tau )\right] ^{\frac{1}{\tau }-1} = \frac{1}{\tau }\left[ \left( 1-\frac{1}{2N_{eE}}\right) ^\tau - II_\infty (\tau ) \right] ^{\frac{1}{\tau }-1}. \end{aligned}$$

Equating the derivative with respect to $\tau$ to 0, of a simplified version of (81) (where $II_\infty (\tau )$ is replaced by $II_\infty$), and assuming $N_{eV}$ is large, it can be shown that

82 $$\begin{aligned} \tau _{\textrm{opt},V} \approx d_{\textrm{opt},V} \cdot 2N_{eE} \end{aligned}$$

whenever $II_\infty \ge 0$, where $d_{\textrm{opt},V} = d_{\textrm{opt},V}(II_\infty )$ solves the equation

83 $$\begin{aligned} d_{\textrm{opt},V} + II_\infty e^{d_{\textrm{opt},V}} = 1. \end{aligned}$$

We interpret $II_\infty$ as a number that quantifies how much migration between subpopulations impacts the variance of allele frequency change. It can be seen from (83) that $d_{\textrm{opt},V}$ is a decreasing function of $II_\infty$, with $d_{\textrm{opt},V}=1$ for $N_{eVMeta}^{\textrm{eq}}$ and $II_\infty = 0$, whereas $d_{\textrm{opt},V}\rightarrow 0$ as $II_\infty \rightarrow 1$. It follows from (83) that $d_{\textrm{opt},V}<\log (II_\infty ^{-1})$, and consequently, the optimal interval (76) is shorter than the length $\tau _{\textrm{max},V}$ of the maximal interval in (69) for which $N_{eV}^{\textrm{eq}}$ exists.

**Fig. 2:**
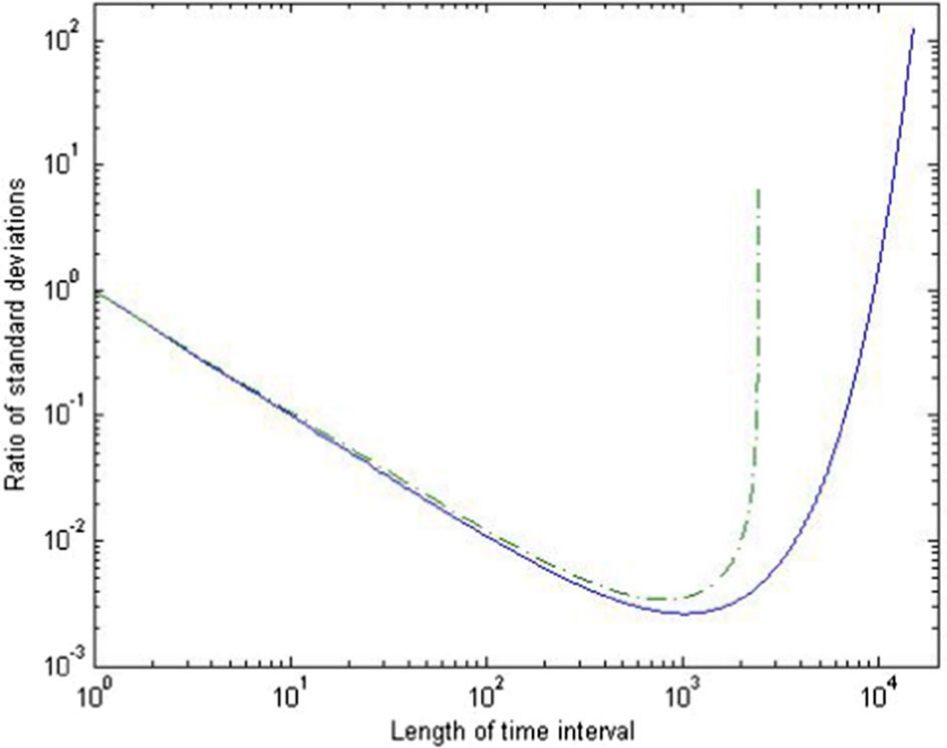
Using the forward definition of variance effective size, the figure shows a log–log plot of the normalized standard deviation $\text{ Var }(\hat{Q})^{1/2}/\sigma$ for estimating $Q=1/(2N_{eV}^{\textrm{eq}})$, the average amount of genetic drift per generation at equilibrium, for time intervals $[0,\tau ]$. The population model is the same as in Fig. 1, and the solid and dash-dotted curves correspond to $N_{eVRMeta}^{\textrm{eq}}$ and $N_{eVRx}^{\textrm{eq}}$ respectively. The solid curve has $\tau _{1.5}=9980$, $\tau _2=10313$, $\tau _3=10780$, $\tau _4=11110$, and $\tau _5=11365$ (cf. (77)), whereas the optimal interval has length $\tau _{\textrm{opt}}=1038$ (cf. (76)). For comparison, formula (83) gives $d_{\textrm{opt}}=1.000$ and $d_{\textrm{opt}}2N_{eE}=1038.6$. The corresponding values of the dash-dotted curve are $\tau _{1.5}=2428$, $\tau _2=2429$, $\tau _3=\tau _4=2430$, $\tau _5=2431$, and $\tau _{\textrm{opt}}=819$, $d_{\textrm{opt}}=0.7886$, and $d_{\textrm{opt}}2N_{eE}=819.0$

Figure 2 is a log–log plot of the normalized standard deviation $\text{ Var }(\hat{Q})^{1/2}/\sigma$ of $\hat{Q}$ as a function of $\tau$ for an island model with $s=10$ subpopulations, when estimating the variance effective size of the metapopulation and a local population respectively. The linear decay to the left of the figure, for smaller $\tau$, corresponds to $\text{ Var }(\hat{Q})^{1/2}$ being inversely proportional to $\tau$ for intervals of short length, in agreement with (75). Note in particular the vertical asymptote of the dash-dotted curve. This corresponds to the fact that $\text{ Var }(\hat{Q})$ diverges when $\tau$ approaches the length of intervals for which $N_{eVRx}^{\textrm{eq}}$ is no longer defined (cf. Fig. 1).

#### Intermediate Approach

For the intermediate definition of the variance effective size we proceed similarly as in Sect. 8.2.1. It follows from (25), (28), and (46) and (47) that

84 $$\begin{aligned} Q(\tau ) = \frac{1 - \big (1-\frac{1}{2N_{eE}}\big )^\tau + II_\infty (\tau )}{1+\frac{1}{2}II_\infty (\tau )}, \end{aligned}$$

which leads to

85 $$\begin{aligned} \frac{dh(Q(\tau ))}{dQ(\tau )} = \frac{1}{\tau }\left[ 1-Q(\tau )\right] ^{\frac{1}{\tau }-1} = \frac{1}{\tau }\left[ \frac{\big (1-\frac{1}{2N_{eE}}\big )^\tau - \frac{1}{2}II_\infty (\tau )}{1+\frac{1}{2}II_\infty (\tau )}\right] ^{\frac{1}{\tau }-1}. \end{aligned}$$

Replacing $II_\infty (\tau )$ in (85) by $II_\infty$ and minimizing with respect to $\tau$, it follows that

86 $$\begin{aligned} \tau _{\textrm{opt},V}^{\textrm{int}} \approx d_{\textrm{opt},V}^{\textrm{int}}\cdot 2N_{eE}, \end{aligned}$$

with

87 $$\begin{aligned} d_{\textrm{opt},V}^{\textrm{int}} + \frac{1}{2}II_\infty e^{d_{\textrm{opt},V}^{\textrm{int}}} = 1. \end{aligned}$$

Notice that $d_{\textrm{opt}}^{\textrm{int},V}=1$ when $II_\infty =0$, whereas $d_{\textrm{opt}}^{\textrm{int},V}<1$ is larger than (83) whenever $II_\infty >0$. This verifies that it is possible to estimate the variance effective size with high accuracy over longer time intervals when the intermediate approach is used, compared to using the forward approach.

**Fig. 3:**
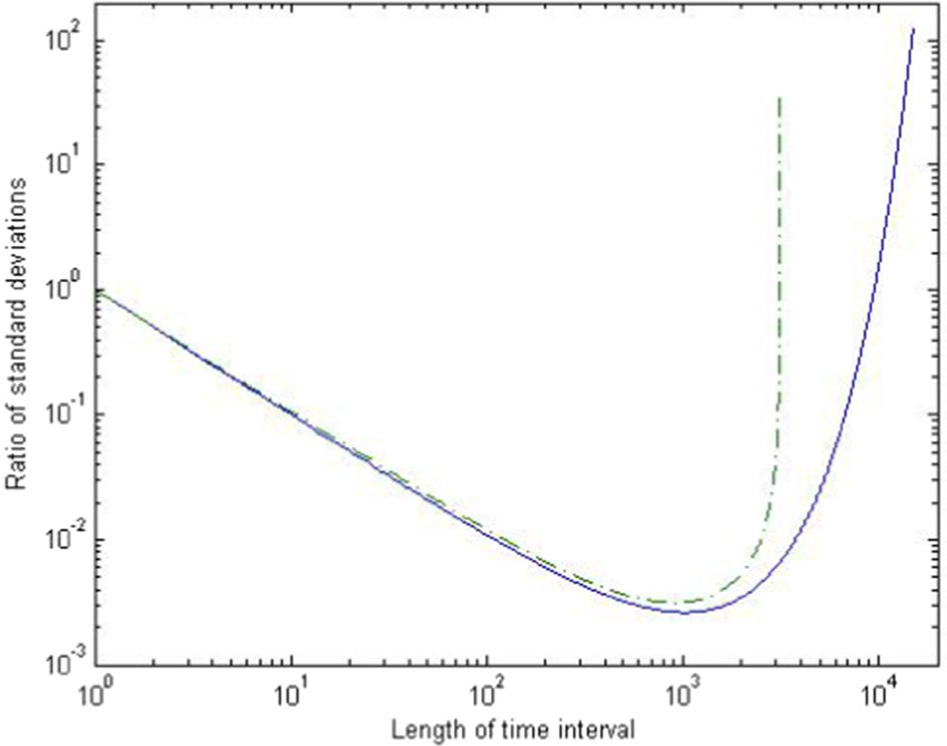
Using the intermediate definition of variance effective size, the figure shows a log–log plot of the normalized standard deviation $\text{ Var }(\hat{Q})^{1/2}/\sigma$ for estimating $Q=1/(2N_{eV}^{\textrm{int,eq}})$, the average amount of genetic drift per generation at equilibrium, for time intervals $[0,\tau ]$. The population model is the same as in Fig. 1, and the solid and dash-dotted curves correspond to $N_{eVRMeta}^{\textrm{int,eq}}$ and $N_{eVRx}^{\textrm{int,eq}}$ respectively. The solid curve has $\tau _{1.5}=9980$, $\tau _2=10313$, $\tau _3=10780$, $\tau _4=11110$, and $\tau _5=11365$ (cf. (77)), whereas the optimal interval has length $\tau _{\textrm{opt}}=1038$ (cf. (76)). For comparison, formula (83) gives $d_{\textrm{opt}}=1.000$ and $d_{\textrm{opt}}2N_{eE}=1038.6$. The corresponding values of the dash-dotted curve are $\tau _{1.5}=3146$, $\tau _2=3147$, $\tau _3=3148$, $\tau _4=\tau _5=3149$, and $\tau _{\textrm{opt}}=917$, $d_{\textrm{opt}}=0.8837$, and $d_{\textrm{opt}}2N_{eE}=917.8$

Figure 3 illustrates $\text{ Var }(\hat{Q})^{1/2}/\sigma$ for an island model with $s=10$ subpopulations. The linear decay to the left of the figure, for smaller $\tau$, corresponds to $\text{ Var }(\hat{Q})^{1/2}$ being inversely proportional to $\tau$ for intervals of short length, in agreement with (75). The vertical asymptote of the dash-dotted curve corresponds to the fact that $\text{ Var }(\hat{Q})$ diverges when $\tau$ approaches the length of intervals for which $N_{eVRx}^{\textrm{int,eq}}$ is no longer defined (cf. Fig. 1).

#### Backward Approach

In order to find $Q(\tau )$ for the backward definition of the variance effective size we combine (30), (33), and (46) and (47). This leads to

88 $$\begin{aligned} Q(\tau ) = \frac{1-\big (1-\frac{1}{2N_{eE}}\big )^{\tau }+II_\infty (\tau )}{1+II_\infty (\tau )} \end{aligned}$$

and

89 $$\begin{aligned} \frac{dh(Q(\tau ))}{dQ(\tau )} = \frac{1}{\tau }\left[ 1 - Q(\tau )\right] ^{\frac{1}{\tau }-1} = \frac{1}{\tau }\frac{\big (1-\frac{1}{2N_{eE}}\big )^{1-\tau }}{(1+II_\infty (\tau ))^{\frac{1}{\tau }-1}}. \end{aligned}$$

It can be seen that the length $\tau _{\textrm{opt},V}^{\textrm{back}}= d_{\textrm{opt},V}^{\textrm{back}} \cdot 2N_{eE}$ that minimizes (89) is proportional to $2N_{eE}$, as in (76). For large $N_{eE}$, the proportionality constant is $d_{\textrm{opt},V}^{\textrm{back}} \approx 1$. Consequently, the length of the optimal interval for the backward version of the variance effective size is

90 $$\begin{aligned} \tau _{\textrm{opt},V}^{\textrm{back}}\approx 2N_{eE}, \end{aligned}$$

similarly as for the gene diversity effective size in (79). Comparing (90) with (83) and (87) we find that the optimal time interval of the backward approach is longer than the corresponding optimal intervals of the forward and intermediate definitions of the variance effective size.

**Fig. 4:**
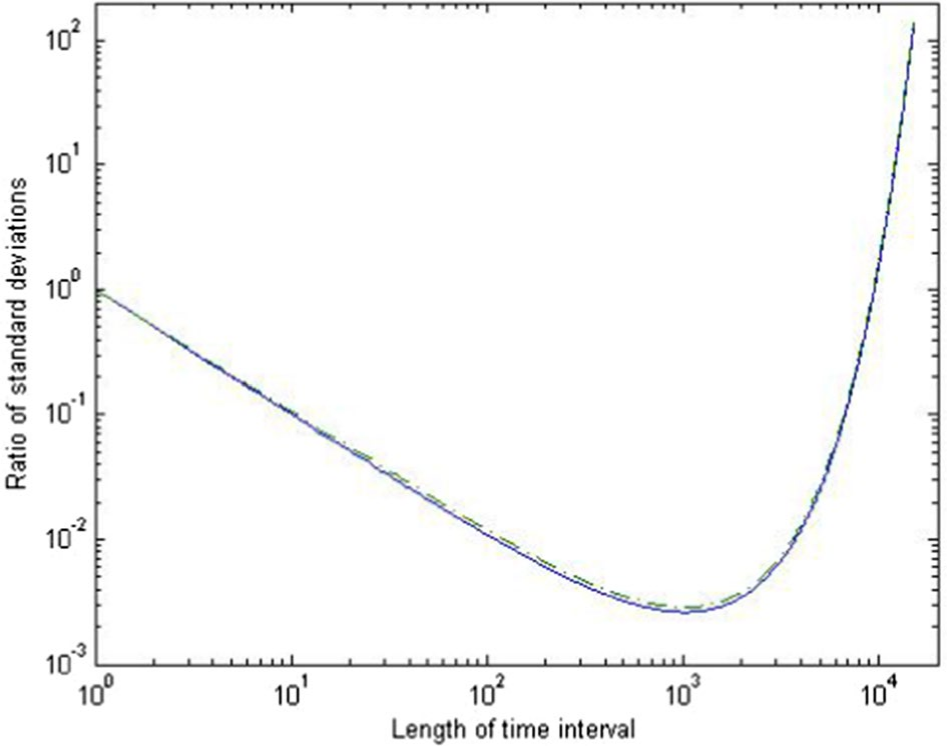
Using the backward definition of variance effective size, the figure shows a log–log plot of the normalized standard deviation $\text{ Var }(\hat{Q})^{1/2}/\sigma$ for estimating $Q=1/(2N_{eV}^{\textrm{back,eq}})$, the average amount of genetic drift per generation at equilibrium, for time intervals $[0,\tau ]$. The population model is the same as in Fig. 1, and the solid and dash-dotted curves correspond to $N_{eVRMeta}^{\textrm{back,eq}}$ and $N_{eVRx}^{\textrm{back,eq}}$ respectively. The solid curve has $\tau _{1.5}=9980$, $\tau _2=10313$, $\tau _3=10780$, $\tau _4=11110$, and $\tau _5=11365$ (cf. (77)), whereas the optimal interval has length $\tau _{\textrm{opt}}=1038$ (cf. (76)). The corresponding values of the dash-dotted curve are $\tau _{1.5}=9874$, $\tau _2=10207$, $\tau _3=10674$, $\tau _4=11005$, $\tau _5=11260$, and $\tau _{\textrm{opt}}=1038$. For comparison, formula (83) gives $d_{\textrm{opt}}=1.000$ and $d_{\textrm{opt}}2N_{eE}=1038.6$ for both curves

Figure 4 illustrates $\text{ Var }(\hat{Q})^{1/2}/\sigma$ for an island model with $s=10$ subpopulations. The linear decay to the left of the figure, for smaller $\tau$, corresponds to $\text{ Var }(\hat{Q})^{1/2}$ being inversely proportional to $\tau$ for intervals of short length, in agreement with (75).

## Estimation of Variance Effective Size from a Real Data Set

In order to illustrate how the variance effective size depends on the chosen subpopulation weights, in this section we analyze a genetic data set of brown trout (*Salmo trutta*) from the Swedish lake Ånnjön. This data set is part of a large longitudinal study comprising 27 different lakes that are located in protected areas of Jämtland County in the central part of Sweden (cf. Andersson et al. (2022) for more details). Biallelic markers are sampled from 96 distinct loci, scattered along the whole genome (all 40 chromosomes) of brown trout, at two time points, corresponding to data that was collected in 1976 and 2017 respectively. Using estimates of the generation time of brown trout, it is assumed that these two time points are approximately $\tau =6$ generations apart. The Structure software (v.2.3.4; Pritchard et al. 2000; Falush et al. 2003) was used to identify $s=3$ cryptic subpopulations within Ånnjön.

The variance effective size is estimated as in Jorde and Ryman (2007). This estimator is defined for a homogeneous population. Its properties for subdivided populations, where the allele frequency at each time point and locus is a weighted average of allele frequencies from all subpopulations, were studied in Ryman et al. (2014, 2023). As mentioned in Sect. 5.3.2, the JR07-estimator targets the intermediate version $N_{eV}^{\textrm{int}}$ of the variance effective size. We will write $\hat{N}_{eV\varvec{w}\varvec{v}}^{\textrm{int}}$ to denote the version of the JR07-estimator that makes use of subpopulation weights $\varvec{w}$ and $\varvec{v}$ at the two time points at which data was collected. It is based on estimates

91 $$\begin{aligned} \begin{array}{rcl} \hat{p}_{tl} &{}=&{} \sum _{x=1}^3 w_x \hat{p}_{txl},\\ \hat{p}_{t+\tau ,l} &{}=&{} \sum _{x=1}^3 v_x \hat{p}_{t+\tau ,xl}, \end{array} \end{aligned}$$

of the subpopulation weighted allele frequencies at loci $l=1,\ldots ,L=96$ and time points *t* and $t+\tau =t+6$ respectively. Here $\hat{p}_{txl}$ refers to the estimated allele frequency at locus *l* in subpopulation *x* at time *t*, based on a sample of size $n_{tx}$. In order to define $\hat{N}_{eV\varvec{w}\varvec{v}}^{\textrm{int}}$ we also need to introduce sample sizes $n_t$ and $n_{t+\tau }$ at time *t* and $t+\tau$. To this end, we use subpopulation weighted harmonic averages

92 $$\begin{aligned} \begin{array}{rcl} n_t &{}=&{} 1/\sum _{x=1}^3 (w_x^2/n_{tx}),\\ n_{t+\tau } &{}=&{} 1/\sum _{x=1}^3 (v_x^2/n_{t+\tau ,x}) \end{array} \end{aligned}$$

of the subpopulation specific sample sizes. The rationale for (92) is that $\text{ Var }(\hat{p}_{txl})=p_{txl}(1-p_{txl})/(2n_{tx})$, and therefore the variances

$$\begin{aligned} \begin{array}{rcl} \text{ Var }(\hat{p}_{tl}) &{}= &{} \sum _x w_x^2 \text{ Var }(\hat{p}_{txl}) \approx p_{tl}(1-p_{tl})/(2n_t),\\ \text{ Var }(\hat{p}_{t+\tau ,l}) &{}= &{} \sum _x v_x^2 \text{ Var }(\hat{p}_{t+\tau ,xl}) \approx p_{t+\tau ,l}(1-p_{t+\tau ,l})/(2n_{t+\tau }), \end{array} \end{aligned}$$

of the estimated subpopulation weighted allele frequencies, in (91), are approximately the same as for homogeneous, binomial samples of sizes $n_t$ and $n_{t+\tau }$.

Table 4 illustrates values of $\hat{N}_{eV\varvec{w}\varvec{v}}^{\textrm{int}}$ for various choices of subpopulation weights $\varvec{w}$ and $\varvec{v}$. In particular, the local realized variance effective sizes $\hat{N}_{eVR1}^{\textrm{int}}=155$, $\hat{N}_{eVR2}^{\textrm{int}}=590$, and $\hat{N}_{eVR3}^{\textrm{int}}=343$ correspond to choosing local weights $\varvec{w}=\varvec{v}=\varvec{e}_x$ for $x=1,2,3$. It can be seen that the intermediate version of the variance effective population size is maximized for local weights of subpopulation 2, i.e. $\hat{N}_{eV\varvec{w}\varvec{v}}^{\textrm{int}}=590$, for $\varvec{w}=\varvec{v}=\varvec{e}_2=(0,1,0)$.

**Table 4:** Estimated variance effective sizes $\hat{N}_{eV\varvec{w}\varvec{v}}^{\textrm{int}}$, based on subpopulation weights $\varvec{w}$ and $\varvec{v}$ at time points *t* and $t+6$, for the brown trout data set of lake Ånnjön, with $s=3$ cryptic subpopulations

Subpopulation weight scenario			$\hat{N}_{eV\varvec{w}\varvec{v}}^{\textrm{int}}$
Type	$\varvec{w}$	$\varvec{v}$	
Sample sizes	$(n_{t1},n_{t2},n_{t3})/C_1$	$(n_{t+6,1},n_{t+6,2},n_{t+6,3})/C_2$	207
Equal	(0.333,0.333,0.333)	$=\varvec{w}$	335
Mostly 1	(1.000,0.000,0.000)	$=\varvec{w}$	155
	(0.833,0.083,0.083)	$=\varvec{w}$	210
	(0.667,0.167,0.167)	$=\varvec{w}$	260
Mostly 2	(0.000,1.000,0.000)	$=\varvec{w}$	590
	(0.003,0.993,0.003)	$=\varvec{w}$	575
	(0.017,0.967,0.017)	$=\varvec{w}$	526
	(0.033,0.933,0.033)	$=\varvec{w}$	479
	(0.083,0.833,0.083)	$=\varvec{w}$	398
	(0.167,0.667,0.167)	$=\varvec{w}$	349
Mostly 3	(0.000,0.000,1.000)	$=\varvec{w}$	343
	(0.017,0.017,0.967)	$=\varvec{w}$	369
	(0.033,0.033,0.933)	$=\varvec{w}$	392
	(0.083,0.083,0.833)	$=\varvec{w}$	436
	(0.167,0.167,0.667)	$=\varvec{w}$	422
Mostly 1 and 2	(0.458,0.458,0.083)	$=\varvec{w}$	337
	(0.417,0.417,0.167)	$=\varvec{w}$	327
Mostly 1 and 3	(0.458,0.083,0.458)	$=\varvec{w}$	263
	(0.417,0.167,0.417)	$=\varvec{w}$	299
Mostly 2 and 3	(0.083,0.458,0.458)	$=\varvec{w}$	413
	(0.167,0.417,0.417)	$=\varvec{w}$	380

For the first subpopulation weight scenario, the weights are proportional to sample sizes, with $C_1=\sum _{x=1}^3 n_{tx}$ and $C_2=\sum _{x=1}^3 n_{t+6,x}$. For all other scenarios, the same subpopulation weights are used at both time points ($\varvec{w}=\varvec{v}$). The (locus averaged) sample sizes at the first time point are $n_{t1}=30$, $n_{t2}=9.9$, and $n_{t3}=9$, whereas at the second time point they are $n_{t+6,1}=19.5$, $n_{t+6,2}=9.7$, and $n_{t+6,3}=19.9$. The realized variance effective sizes $\hat{N}_{eVR1}^{\textrm{int}} = 155$, $\hat{N}_{eVR2}^{\textrm{int}} = 590$, and $\hat{N}_{eVR3}^{\textrm{int}} = 343$ for subpopulations 1,2,3 correspond to values of $\hat{N}_{eV\varvec{w}\varvec{v}}^{\textrm{int}}$ for the three local weighting schemes $\varvec{w}=\varvec{v}=\varvec{e}_x$, for $x=1,\;2,\;3$

Recall the discussion of Sect. 6.4.2 that equations (55) and (56) are also valid for the intermediate version of the variance effective size, if the system is in migration–drift equilibrium. The findings of Table 4 could therefore indicate that the reproductive weights $\varvec{\gamma }$ are close to $\varvec{e}_2$, so that $N_{eVMeta}^{\textrm{eq}}=N_{eE}$ is close to $\hat{N}_{eV\varvec{e}_2}^{\textrm{int}}=590$. According to Sect. 3, $\gamma =(\gamma _1,\gamma _2,\gamma _3)$ contains the long term genetic contributions from the three subpopulations. If our conclusion $\gamma _2\approx 1$ is correct, this indicates that $x=2$ is a source population from which most or all genetic material originates (i.e. unidirectional migration from 2 to 1 and 3). However, for at least two reasons, this is so far only a conjecture: Firstly, more data analysis, with larger sample sizes and more loci, is needed in order to confirm the conclusion that 2 is a source population. Although the JR07-estimator corrects for the sampling effect, the low sample sizes (for $x=2$ in particular) of this data set indicate that the results of Table 4 are a bit uncertain. A separate analysis, based on the (wrong) assumption that all sample sizes are very large, gives a maximal variance effective size $\hat{N}_{eV\varvec{w}\varvec{v}}^{\textrm{int}}$ when the three subpopulations are weighted close to uniformly ($w_x=v_x\approx 1/3$ for $x=1,2,3$) at both time points, with a corresponding much lower value of $N_{eE}$. Secondly, the theoretical results (55) and (56) have only been proved for populations in migration–drift equilibrium, with (55) derived for island models and (56) for models with symmetric migration between subpopulations.

## Discussion

In this paper we study the variance effective size $N_{eV}$ of a substructured population, with particular focus on the size of the metapopulation ($N_{eVMeta}$). Our main findings are: (i) That the version of $N_{eV}$ that is of interest for conservation, under certain conditions can be found by maximizing the variance effective size with respect to subpopulation weights in order to minimize the impact of migration and approximate $N_{eGD}$, (ii) that two new and more stable versions of $N_{eV}$ are introduced and (iii) that the length of the optimal time window of $N_{eV}$, in terms of estimation accuracy, is derived.

As a major tool for understanding the properties of $N_{eV}$, we analyze in detail two components of expected squared allele frequency change, defined in equations (21)–(23). The first term *I* is caused by genetic drift in subpopulations between the two time points at which genetic data is collected, whereas the second term *II* (or more precisely $-II$) quantifies a correlation between allele frequency change of the past and present. We refer to *II* as a migration or gene flow term, since it is mainly caused by gene flow between subpopulations, when these are assigned the same weights at both time points at which allele frequencies are estimated from data. General expressions are obtained for how the genetic drift and gene flow terms *I* and *II* involve the local census sizes and local effective sizes of subpopulations, the migration pattern between subpopulations and the way in which subpopulations are weighed at the two time points between which genetic change is monitored.

The variance effective size is traditionally defined as in (20), so that expected squared allele frequency change is normalized by its expectation, a normalization that involves allele frequencies at the first time point at which genetic data is collected. We refer to this as the forward version of $N_{eV}$, since it corresponds to a forward time perspective on how allele frequency change is normalized. As mentioned under (ii), in this article we also introduce, in (25) and (30), two other notions of variance effective size, the intermediate and backward versions of $N_{eV}$, for which allele frequency change is normalized based on expected allele frequency change at both or only the last time point at which genetic data is collected.

The abovementioned three versions of $N_{eV}$ are very close when the interval between the two time points at which genetic data is collected is small, but they start to differ substantially for intervals with a length that is at least of the same order as the eigenvalue effective size $N_{eE}$. Two numerical examples are given in this paper in order to illustrate this. The first example represents a large metapopulation with $s=10$ subpopulations of size 50, for which 200–300 generations are required for the three versions of $N_{eV}$ to differ substantially. The second example represents a small metapopulation with $s=2$ subpopulations of size 10, for which less than 10 generations is sufficient for the three versions of $N_{eV}$ to differ significantly. We also show that the backward version of $N_{eV}$ is most stable and exists under general conditions, for time intervals of any length. In addition, as mentioned under (iii), we derive in (76), (86), and (90) the length of the optimal time interval for which the forward, intermediate and backward versions of $N_{eV}$ are estimated with maximal accuracy.

As mentioned under (i), a major implication of our work is that the variance effective size of a substructured population, with appropriately chosen subpopulation weights, is relevant for conservation applications. In more detail, let $\hat{N}_{eV\varvec{w}}$ be an estimate of the variance effective size, based on using the same subpopulation weight vector $\varvec{w}=\varvec{v}$ at both time points at which genetic data is collected. We conjecture that

93 $$\begin{aligned} \hat{N}_{eE} = \max _{\varvec{w}} \hat{N}_{eV\varvec{w}} \end{aligned}$$

is an estimate of the eigenvalue effective size $N_{eE}$ for some population systems close to migration–drift equilibrium. The rationale for (93) is equation (56), which implies that the variance effective size $N_{eV\varvec{w}}$, based on using the same subpopulation weights $\varvec{w}=\varvec{v}$ at both time points at which genetic data is collected, is maximized for reproductive subpopulation weights $\varvec{w}=\varvec{\gamma }$. This follows from the fact that $N_{eV\varvec{w}}$ is maximized when the gene flow term *II* vanishes, which happens for reproductive subpopulation weights $\varvec{\gamma }$. The conservation relevance of (93) follows from the fact that (a) $N_{eV\varvec{\gamma }}$ is closely related to the gene diversity effective size $N_{eGD}$, (b) $N_{eGD}$ equals $N_{eE}$ under migration drift equilibrium, and (c) $N_{eGD}$ also approximates the additive genetic variance effective size $N_{eAV}$, which is of particular interest for long term conservation (Hössjer et al. 2016). Because of the conservation relevance of (93), it is of interest to develop software that automatically perform the maximization of this equation in order to compute $\hat{N}_{eE}$.

The reproductive weights $\varvec{\gamma }$ depend on the migration pattern between the subpopulations, which typically is unknown. However, equation (93) suggests that it is possible to estimate $\varvec{\gamma }$ indirectly (without first estimating migration rates between subpopulations) as the subpopulation weights that maximize $\hat{N}_{eV\varvec{w}}$. If all subpopulations contribute to the long term reproduction of the metapopulation, all components of $\varvec{\gamma }$ are positive. Whenever this is the case, in order to compute the estimator of $N_{eE}$ in (93), it is required that genetic data is collected from all subpopulations at the two time points between which genetic change is monitored. On the other hand, analysis of the dataset in Sect. 9 indicates that one of the subpopulations might be a source, since the maximum of (93) occurs when this subpopulation is assigned a maximal weight of 1. If this is a correct interpretation of the biological situation, only data from this subpopulation is needed in order to estimate $N_{eE}$. However, in order to confirm this conclusion a larger dataset is needed, and the validity of (93) must be investigated beyond our present theoretical assumptions (migration–drift equilibrium and symmetric backward migration rates $B_{xy}=B_{yx}$ between all pairs *x*, *y* of subpopulations, which implies $\varvec{\gamma }=(1/s,\ldots ,1/s)$) fail.

Several extensions of our work are possible. Firstly, it is possible to investigate whether the present conditions (migration–drift equilibrium and symmetric migration) for equations (56) and (93) can be extended to structured populations of more general form.

Secondly, it is of interest to develop a multilocus estimator of the backward version $N_{eV}^{\textrm{back}}$ of the variance effective size, which analogously to the JR07-estimator of $N_{eV}^{\textrm{int}}$ in Jorde and Ryman (2007) adjusts for finite sampling.

Thirdly, for conservation purposes it is important to study the relation between $N_{eV}$, $N_{eGD}$, and $N_{eAV}$ for more general models. We have emphasized that $N_{eV}$, with reproductive subpopulation weights $\varvec{w}=\varvec{v}=\varvec{\gamma }$, is closely related to $N_{eGD}$ and $N_{eAV}$ (and also with $N_{eE}$ under migration–drift equilibrium). However, this is based on the assumption that $N_{eAV}$ refers to the change of additive genetic variance of a quantitative trait with no epistasis (Hössjer et al. 2016). It is therefore of interest to give more general expressions for $N_{eAV}$ when epistasis is taken into account. We conjecture that $N_{eAV}$ is still very similar to $N_{eGD}$, and $N_{eV}$ with reproductive weights, for models with epistasis, since all these three effective sizes only involve the drift term *I*, whereas $N_{eV}$ with other subpopulation weights will be different, since it also involves the correlation term *II* between past and present allele frequency change.

## Figures and Tables

**Fig. 5:**
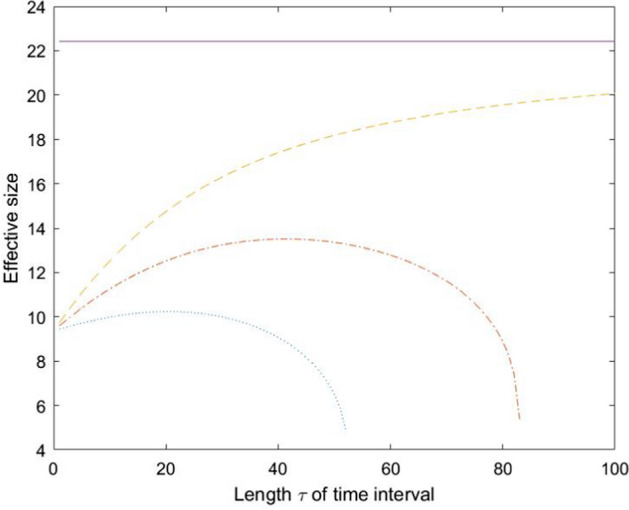
The figure plots effective sizes for an island model with $s=2$, $N_{ex}=N_{cx}=10$ and $m=0.1$, when $t=0$ corresponds to migration–drift equilibrium ($T\rightarrow \infty$). The horizontal solid line depicts $N_{eE}=22.42$. The three curves correspond to $N_{eVRx}$ (dotted), $N_{eVRx}^{\textrm{int}}$ (dash-dotted) and $N_{eVRx}^{\textrm{back}}$ (dashed) for intervals $[0,\tau ]$ of increasing length. $N_{eVRx}$ increases with $\tau$ at first, then it starts to drop until $\tau =\tau _{\textrm{max}}=52$, and for longer intervals $N_{eVRx}$ does not exist. In comparison, formula (69) predicts $\tau _{\textrm{max,V}}=\log (II_\infty ^{-1})\cdot 2N_{eE}= 52.96$. In a similar fashion $N_{eVRx}^{\textrm{int}}$ increases with $\tau$ at first, then it starts to drop until $\tau =\tau _{\textrm{max}}=83$, and after this generation $N_{eVRx}^{\textrm{int}}$ does not exist. In comparison, formula (71) predicts $\tau _{\textrm{max},V}^{\textrm{int}}=\log (2II_\infty ^{-1})\cdot 2N_{eE}= 84.04$. On the other hand, $N_{eVRx}^{\textrm{back}}$ increases monotonically to $N_{eE}$ as $\tau \rightarrow \infty$

**Fig. 6:**
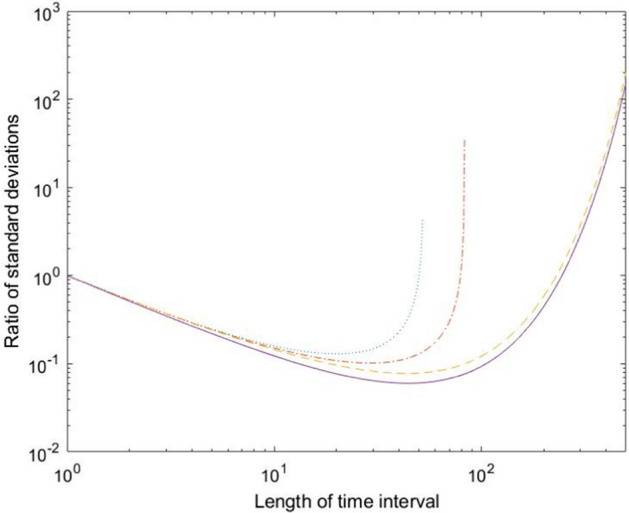
The figure shows a log–log plot of the normalized standard deviation $\text{ Var }(\hat{Q})^{1/2}/\sigma$ for estimating $Q=1/(2N_{eV\varvec{w}}^{\textrm{method,eq}})$, the average amount of genetic drift per generation at equilibrium, for time intervals $[0,\tau ]$. The population is an island model with $s=2$, $N_{ex}=N_{cx}=10$, and $m=0.1$. The dotted, dashed and dashdotted lines correspond to variance effective sizes of a local population ($\varvec{w}=x$), using the forward, intermediate and backward methods respectively, whereas the solid line (the same for all three methods) corresponds to the metapopulation ($\varvec{w}=\text{ Meta }$). The optimal time intervals have lengths $\tau _{\textrm{opt}}=20$, $\tau _{\textrm{opt}}^{\textrm{int}}=29$, and $\tau _{\textrm{opt}}^{\textrm{int}}=44$ for the local curves of the forward, intermediate and backward methods, and $\tau _{\textrm{opt,Meta}}=44$ for the metapopulation. The corresponding approximations are $d_{\textrm{opt}}2N_{eE} = 22.24$, $d_{\textrm{opt}}^{\textrm{int}}2N_{eE} = 31.07$, $d_{\textrm{opt}}^{\textrm{back}}2N_{eE} = 44.83$, and $d_{\textrm{opt,Meta}}2N_{eE} = 44.83$ respectively. The largest time intervals for which the forward and intermediate effective sizes can be estimated, are $\tau _{\textrm{max},V}=52$ and $\tau _{\textrm{max},V}^{\textrm{int}}=83$ respectively

## A: Appendix with Numerical Examples for a Small and Subdivided Population

In this appendix we demonstrate that sometimes the forward, intermediate, and backward versions of the local realized variance effective size differ substantially for a small and subdivided population, even for time intervals $[0,\tau ]$ of moderate (and biologically realistic) lengths.

This is illustrated in Fig. 5 for an island model with $s=2$ subpopulations of sizes $N_{ex}=N_{cx}=10$, and a migration rate of $m=0.1$. It can be seen that the three effective size differ a lot already for $\tau =5$, with values $N_{eVRx}=9.71$, $N_{eVRx}^{\textrm{int}}=10.39$, and $N_{eVRx}^{\textrm{back}}=11.07$, and even more for $\tau =10$, with values $N_{eVRx}=9.98$, $N_{eVRx}^{\textrm{int}}=11.25$, and $N_{eVRx}^{\textrm{back}}=12.51$. The forward and intermediate versions of the realized variance effective size exist for intervals of length up to $\tau =52$ and 83 generations respectively, whereas the backward version of the realized variance effective exists for intervals of any length.

The corresponding plot of the accuracy of estimates of genetic drift per generation, for intervals of varying length, also reveals a substantial difference between the three versions of variance effective size (cf. Fig. 6).

## B: Appendix with Further Numerical Examples and Proofs

### B.1: Series Expansion of $\varvec{r}$

We will view the right eigenvector $\varvec{r}=\varvec{r}(\varepsilon )$ of $\varvec{A}$ as a function of $\varepsilon =1/(2N_{eE})$. Assuming that $0<N_{eE}\le \infty$ is large, or equivalently that $\varepsilon \ge 0$ is small, we apply perturbation theory of matrices (Maruyama 1970a; Nagylaki 1980, 1995; Hössjer 2015) in order to find a linear approximation $\varvec{r}(\varepsilon ) \approx \varvec{r}(0) + \dot{\varvec{r}}\varepsilon$.

Put $e_x=N_{eE}/N_{ex}$, $c_x=N_{eE}/N_{cx}$ and rewrite the elements of $\varvec{A}=\varvec{A}(\varepsilon )$ in (4) as

$$\begin{aligned} \begin{array}{rcl} A_{xy,zu}(\varepsilon ) &{}=&{} (1-c_x\varepsilon )^{1(x=y)} B_{xz}B_{yu}[(1-e_x\varepsilon )/(1-c_x\varepsilon )]^{1(z=u)}\\ &{}\approx &{} A_{xz,yu}(0) + \varepsilon \dot{A}_{xz,yu}, \end{array} \end{aligned}$$

where

94 $$\begin{aligned} A_{xy,zu}(0) = B_{xz}B_{yu} \end{aligned}$$

and

95 $$\begin{aligned} \dot{A}_{xy,zu} = \left[ (c_z-e_z)1(z=u) - c_x 1(x=y)\right] B_{xz}B_{yu}. \end{aligned}$$

Note in particular that the two exponents $1(x=y)$ and $1(z=u)$ appear in $A_{xy,zu}(\varepsilon )$ as a consequence of (4). For this reason $A_{xy,zu}(\varepsilon )$ varies with $\varepsilon$, and $\dot{A}_{xy,zu}\ne 0$, only when at least one of the two conditions $x=y$ and $z=u$ is satisfied. It follows from (94) that $\varvec{r}(0)=\varvec{1}_{s^2}=\varvec{1}$ is a right eigenvector of $\varvec{A}(0)$ with eigenvalue 1, and therefore

96 $$\begin{aligned} \varvec{r}(\varepsilon ) \approx \varvec{1}+ \varepsilon \dot{\varvec{r}}+ o(\varepsilon ) \end{aligned}$$

is a valid first order Taylor approximation of $\varvec{r}(\varepsilon )$. In order to find a more explicit expression of $\dot{\varvec{r}}$, we will rewrite it as a linear combination of a system of orthonormal basis functions. To this end, recall that $\varvec{l}_i = (l_{i1},\ldots ,l_{is})$ is a left eigenvector of $\varvec{B}$ with a real-valued eigenvalue $\eta _i$, and that $\{\varvec{l}_i\}_{i=1}^s$ is an orthonormal system of basis functions for ${\mathbb {R}}^s$. Define, for each $1\le i,j \le s$ a row vector $\varvec{l}_{ij} = (l_{ij,xy} = l_{ix}l_{jy}; 1\le x,y \le s)$ of length $s^2$. Then $\{\varvec{l}_{ij}; \, 1\le i,j \le s\}$ forms an orthonormal system of basis functions for ${\mathbb {R}}^{s^2}$. We also introduce

97 $$\begin{aligned} \xi _{ij} = \varvec{l}_{ij}\dot{\varvec{r}}= \sum _{xy} l_{ij,xy}\dot{r}_{xy} \end{aligned}$$

as the coefficient of $\varvec{l}_{ij}^T$ in the basis function expansion

98 $$\begin{aligned} \dot{\varvec{r}}= \sum _{ij} \xi _{ij}\varvec{l}_{ij}^T \end{aligned}$$

of $\dot{\varvec{r}}$. Since $\varvec{l}_{11}=\varvec{1}/s$ is proportional to $\varvec{r}(0)=\varvec{1}$, we may assume that a change from $\varepsilon =0$ to $\varepsilon >0$ results in a perturbation $\varvec{r}(\varepsilon )-\varvec{r}(0)$ orthogonal to $\varvec{r}(0)$. This corresponds to an assumption $0=\xi _{11}=\varvec{l}_{11}\dot{\varvec{r}}= \varvec{1}^T\dot{\varvec{r}}/s$. In order to find a more explicit expression for all $\{\xi _{ij}; \, (i,j)\ne (1,1)\}$, we will derive a linear system of equations for the components of $\dot{\varvec{r}}=(\dot{r}_{xy})$, based on an analysis of how $\varvec{r}(\varepsilon )$ is perturbed after small change of $\varepsilon$ away from zero. From the definition of $\varepsilon$, and of the eigenvalue effective size in (34), it follows that $\lambda (\varepsilon ) = 1 - \varepsilon$. Together with (96), this makes it possible to rewrite $\lambda (\varepsilon )\varvec{r}(\varepsilon )=\varvec{A}(\varepsilon )\varvec{r}(\varepsilon )$ as

99 $$\begin{aligned} \begin{array}{rcl} (1-\varepsilon )(\varvec{1}+ \varepsilon \dot{\varvec{r}}) &{}=&{} (\varvec{A}(0) + \varepsilon \dot{\varvec{A}}) (\varvec{1}+ \varepsilon \dot{\varvec{r}}) + o(\varepsilon )\\ &{}=&{} \varvec{1}+ \varepsilon (\dot{\varvec{A}}\varvec{1}+ \varvec{A}(0)\dot{\varvec{r}}) + o(\varepsilon ). \end{array} \end{aligned}$$

Equating the linear $\varepsilon$-terms of the left- and right-hand sides of (99) we find, after some rearrangements, that

100 $$\begin{aligned} \dot{\varvec{r}}- \varvec{A}(0)\dot{\varvec{r}}= \varvec{1}+ \dot{\varvec{A}}\varvec{1}. \end{aligned}$$

Because of (94) and (95), component *xy* of Eq. (100) takes the form

101 $$\begin{aligned} \begin{array}{rcl} \dot{r}_{xy} - \sum _{z,u} B_{xz}B_{yu}\dot{r}_{zu} &{}=&{} 1 + (\dot{\varvec{A}}\varvec{1})_{xy}\\ &{}=&{} 1 - c_x 1(x=y) + \sum _{z} (c_z-e_z)B_{xz}B_{yz}\\ &{}=&{} 1 - c1(x=y) + (c-e) \sum _{z} B_{xz}B_{yz}, \end{array} \end{aligned}$$

where the term $1(x=y)$ of the second step is inherited from (95). In the last step of (101) we assumed $N_{ex}=N_e$ and $N_{cx}=N_c$, so that $e_x=e$ and $c_x=c$. Assume that $(i,j)\ne (1,1)$. Multiplying the left and right hand sides of (101) with $l_{ij,xy}$, and summing jointly over *x* and *y*, we find, making use of

$$\begin{aligned} \begin{array}{rcl} \sum _{x,y}l_{ij,xy}\dot{r}_{xy} &{}=&{} \xi _{ij},\\ \sum _{x,y} l_{ij,xy}B_{xz}B_{yu} &{}=&{} \eta _i\eta _j l_{ij,zu}, \\ \sum _{x,y} l_{ij,xy}B_{xz}B_{yz} &{}=&{} \eta _i \eta _j l_{ij,zz}, \\ \sum _{x,y} l_{ij,xy}1(x=y) &{}=&{} \sum _{x} l_{ij,xx} = 1(i=j), \end{array} \end{aligned}$$

that

$$\begin{aligned} (1-\eta _i\eta _j)\xi _{ij} = - c 1(i=j) + (c-e)\eta _i^2 1(i=j), \end{aligned}$$

or equivalently

102 $$\begin{aligned} \xi _{ij} = \left\{ \begin{array}{ll} 1(i=j) \cdot (- c + (c-e)\eta _i^2)/(1-\eta _i^2), &{} (i,j)\ne (1,1),\\ 0, &{} (i,j)=(1,1). \end{array}\right. \end{aligned}$$

### B.2: Proof of Equation (43)

Inserting (96) into (42) we find that

103 $$\begin{aligned} \begin{array}{rcl} F_{ST}^{\textrm{eq}} &{}\approx &{} \left( \sum _{x,y} w_x w_y \dot{r}_{xy} - \sum _x w_x\dot{r}_x\right) \varepsilon \\ &{}=&{} - s^{-1}\sum _x \dot{r}_x \cdot \varepsilon \\ &{}=&{} - s^{-1}\sum _{ij} \xi _{ij} \varvec{l}_i\varvec{l}_j^T \cdot \varepsilon \\ &{}=&{} s^{-1}\sum _{i=2}^s [c-(c-e)\eta _i^2]/(1-\eta _i^2) \cdot \varepsilon \\ &{}=&{} s^{-1}\sum _{i=2}^s [1/N_c - (1/N_c - 1/N_e)\eta _i^2]/[2(1-\eta _i^2)], \end{array} \end{aligned}$$

where in the second step of (103) we used $\varvec{w}=\varvec{\gamma }=\varvec{1}_s/s$ and the fact that $\sum _{xy} \dot{r}_{xy} = \xi _{11}=0$. In the third step of (103) we inserted the series expansion (98) of $\dot{\varvec{r}}$, in the fourth step we utilized (102), and in the final step we invoked the definitions of $c=N_{eE}/N_c$, $e=N_{eE}/N_e$, and $\varepsilon =1/(2N_{eE})$. This completes the proof since the right hand side of (103) agrees with (43).

### B.3: Proof of Equation (48)

In order to prove (48) we will utilize the series expansion of $\varvec{r}$ (or $\dot{\varvec{r}}$). As a first step we need to express $II_\infty (\tau )$ in terms of $\dot{\varvec{r}}$. Insertion of (96) into (47) yields

104 $$\begin{aligned} II_\infty (\tau ) \approx 2\sum _{x,y} \left[ (\varvec{v}\varvec{B}^\tau )_x - w_x \right] w_y \dot{r}_{xy} \cdot \varepsilon . \end{aligned}$$

Since $\kappa _i = \varvec{w}\varvec{l}_i^T$ and $\rho _i=\varvec{v}\varvec{l}_i^T$ by assumption, it follows that the analogue of (104), without the factor 2 and with $l_{ij,xy}$ in place of $\dot{r}_{xy}$, reads

105 $$\begin{aligned} \begin{array}{rcl} \sum _{xy} \left[ (\varvec{v}\varvec{B}^\tau )_x - w_x \right] w_y \cdot l_{ij,xy} &{}=&{} \sum _x \left[ (\varvec{v}\varvec{B}^\tau )_x - w_x \right] l_{ix} \cdot \sum _y w_y l_{jy}\\ &{}=&{} (\eta _i^\tau \rho _i -\kappa _i)\kappa _j. \end{array} \end{aligned}$$

Inserting (98) and (105) into (104) we find that

106 $$\begin{aligned} II_\infty (\tau ) \approx 2 \sum _{(i,j)\ne (1,1)} (\eta _i^\tau \rho _i - \kappa _i)\kappa _j \xi _{ij} \cdot \varepsilon . \end{aligned}$$

Then we insert the expression (102) for $\xi _{ij}$, that was derived in Appendix B.1, into (106). This yields

107 $$\begin{aligned} \begin{array}{rcl} II_\infty (\tau ) &{}\approx &{} 2 \sum _{i=2}^s (\eta _i^\tau \rho _i - \kappa _i)\kappa _i \frac{- c + (c-e)\eta _i^2}{1-\eta _i^2} \cdot \varepsilon \\ &{}=&{} 2 \sum _{i=2}^s \frac{\kappa _i(\kappa _i-\rho _i\eta _i^\tau )}{1-\eta _i^2} \left[ (1-\eta _i^2)c + \eta _i^2 e\right] \cdot \varepsilon . \end{array} \end{aligned}$$

By substituting the definitions of $\varepsilon =1/(2N_{eE})$, $c=N_{eE}/N_c$ and $e=N_{eE}/N_e$ into (107) we finally arrive at (48).

### B.4: Proofs of Equations (55) and (56)

Recall that Eq. (55) stipulates that the variance effective size at equilibrium, for the island model, is maximized when the same subpopulation weights are used at time points *t* and $t+\tau$ ($\varvec{w}=\varvec{v}$). Equation (56), on the other hand, is a claim that whenever the same subpopulation weights are used at both time points ($\varvec{w}=\varvec{v}$), the variance effective size at equilibrium is maximized for reproductive subpopulation weights ($\varvec{w}=\varvec{v}=\varvec{\gamma }$). In order to prove these claims we will make use of (52), which states that the variance effective size $N_{eV}^{\textrm{eq}}$ under equilibrium is a strictly decreasing function of the equilibrium gene flow term $II_\infty (\tau )$ of Eq. (47). That is, we need to translate (55) and (56) into analogous inequalities for the equilibrium gene flow term in (47). To this end, we will highlight that this term is a function of the weight vectors $\varvec{w}$ and $\varvec{v}$ at time points *t* and $t+\tau$. More specifically, we will write $II_\infty (\tau )=II_{\infty \varvec{w}\varvec{v}}(\tau )$ and $II_{\infty \varvec{w}}(\tau )=II_{\infty \varvec{w}\varvec{w}}(\tau )$. Then, in order to prove (55) it suffices to establish that

108 $$\begin{aligned} II_{\infty \varvec{w}\varvec{v}}(\tau ) \ge II_{\infty \varvec{w}}(\tau ) \end{aligned}$$

for the island model whenever $\varvec{w}\varvec{v}^T\le |\varvec{w}|^2$, with equality in (108) if and only if $\varvec{w}\varvec{v}=|\varvec{w}|^2$. And in order to establish (56) it suffices to prove that

109 $$\begin{aligned} II_{\infty \varvec{w}}(\tau ) \ge II_{\infty \varvec{\gamma }}(\tau ) = 0, \end{aligned}$$

for the symmetric migration models of Example 1, with equality if and only if $\varvec{w}=\varvec{\gamma }$. But (108) follows immediately from (49), whereas (109) is deduced from (48) with $\kappa _i=\rho _i$ (since $\varvec{w}=\varvec{v}$ is assumed), and the fact that $\varvec{w}=\varvec{\gamma }$ if and only if $\kappa _2=\ldots =\kappa _s=0$.
